# Rapid remodeling of the soil lipidome in response to a drying-rewetting event

**DOI:** 10.1186/s40168-022-01427-4

**Published:** 2023-02-27

**Authors:** Sneha P. Couvillion, Robert E. Danczak, Dan Naylor, Montana L. Smith, Kelly G. Stratton, Vanessa L. Paurus, Kent J. Bloodsworth, Yuliya Farris, Darren J. Schmidt, Rachel E. Richardson, Lisa M. Bramer, Sarah J. Fansler, Ernesto S. Nakayasu, Jason E. McDermott, Thomas O. Metz, Mary S. Lipton, Janet K. Jansson, Kirsten S. Hofmockel

**Affiliations:** 1 Earth and Biological Sciences Directorate, Pacific Northwest National Laboratory, Richland, WA USA; 2 National Security Directorate, Pacific Northwest National Laboratory, Richland, WA USA; 3 Department of Molecular Microbiology and Immunology, Oregon Health & Science University, Portland, OR USA; 4 Department of Agronomy, Iowa State University, Ames, IA USA

**Keywords:** Soil, Lipidomics, Summer drought, Drying-rewetting, Metabolomics

## Abstract

**Background:**

Microbiomes contribute to multiple ecosystem services by transforming organic matter in the soil. Extreme shifts in the environment, such as drying-rewetting cycles during drought, can impact the microbial metabolism of organic matter by altering microbial physiology and function. These physiological responses are mediated in part by lipids that are responsible for regulating interactions between cells and the environment. Despite this critical role in regulating the microbial response to stress, little is known about microbial lipids and metabolites in the soil or how they influence phenotypes that are expressed under drying-rewetting cycles. To address this knowledge gap, we conducted a soil incubation experiment to simulate soil drying during a summer drought of an arid grassland, then measured the response of the soil lipidome and metabolome during the first 3 h after wet-up.

**Results:**

Reduced nutrient access during soil drying incurred a replacement of membrane phospholipids, resulting in a diminished abundance of multiple phosphorus-rich membrane lipids. The hot and dry conditions increased the prevalence of sphingolipids and lipids containing long-chain polyunsaturated fatty acids, both of which are associated with heat and osmotic stress-mitigating properties in fungi. This novel finding suggests that lipids commonly present in eukaryotes such as fungi may play a significant role in supporting community resilience displayed by arid land soil microbiomes during drought. As early as 10 min after rewetting dry soil, distinct changes were observed in several lipids that had bacterial signatures including a rapid increase in the abundance of glycerophospholipids with saturated and short fatty acid chains, prototypical of bacterial membrane lipids. Polar metabolites including disaccharides, nucleic acids, organic acids, inositols, and amino acids also increased in abundance upon rewetting. This rapid metabolic reactivation and growth after rewetting coincided with an increase in the relative abundance of firmicutes, suggesting that members of this phylum were positively impacted by rewetting.

**Conclusions:**

Our study revealed specific changes in lipids and metabolites that are indicative of stress adaptation, substrate use, and cellular recovery during soil drying and subsequent rewetting. The drought-induced nutrient limitation was reflected in the lipidome and polar metabolome, both of which rapidly shifted (within hours) upon rewet. Reduced nutrient access in dry soil caused the replacement of glycerophospholipids with phosphorus-free lipids and impeded resource-expensive osmolyte accumulation. Elevated levels of ceramides and lipids with long-chain polyunsaturated fatty acids in dry soil suggest that lipids likely play an important role in the drought tolerance of microbial taxa capable of synthesizing these lipids. An increasing abundance of bacterial glycerophospholipids and triacylglycerols with fatty acids typical of bacteria and polar metabolites suggest a metabolic recovery in representative bacteria once the environmental conditions are conducive for growth. These results underscore the importance of the soil lipidome as a robust indicator of microbial community responses, especially at the short time scales of cell-environment reactions.

Video Abstract

**Supplementary Information:**

The online version contains supplementary material available at 10.1186/s40168-022-01427-4.

## Background

Across the globe, intense heatwaves and droughts are becoming more frequent and severe [1, 2]. Inextricably linked, heat can exacerbate drought, which in turn can cause hotter temperatures. These drought events increase moisture and heat stress, with important implications for soil functions [3, 4], including shifts in carbon (C) cycling. Microbial metabolic activity is generally reduced during heat and moisture stress or drought periods, either due to cell death or transformation to a dormant, metabolically inactive state [5]. Upon wetting of dry soils, there is a rapid physiological response of the soil microbiome, most often measured as a burst of respiration (CO_2_) known as the Birch effect [6]. We and others have previously shown that in multiple soils, including the soil from this field site, the CO_2_ production peaks within the first 180 min of rewetting [7, 8]. In dry grassland ecosystems, this pulse can contribute disproportionately to the ecosystem carbon budget [9]. In addition to respiration rates, previous studies [7, 10–15] have investigated the effects of soil drying and rewetting on microbial biomass, community structure, taxon-specific growth rates, substrate use efficiencies, compatible solutes, protein, and gene expression [13, 14, 16]. However, our understanding of how the rewetting of dry soil impacts microbial physiology and metabolism in those first few hours following moisture addition remains unclear. Environmental stress can have interdependent effects on microbial metabolism and community composition. The metabolic response of microbial members to environmental change is important to characterize because it can influence community-level metabolic interactions and dependencies [17] which can shape composition and potentially have ecosystem-scale impacts.

Lipids are generally recognized as being highly responsive to environmental perturbations [18–20] and could play a role in enabling the adaptation of soil microbial communities to drought stress. Soil microorganisms can modulate the biophysical properties of their membranes, such as fluidity, to confer resilience to a changing environment. Changes in lipid composition can initiate and indicate the activation of the cellular stress responses and impact key metabolic pathways required for growth [18, 21, 22]. Cellular lipids can be remodeled to conserve energy and optimize the use of essential nutrients such as C, nitrogen (N), and phosphorus (P) under conditions of nutrient limitation. They can also be degraded and used as endogenous C and energy sources [23]. Therefore, characterizing how the soil lipidome shifts during drought and rewetting should provide valuable knowledge about the physiological response of soil microbes to environmental changes. However, for the vast majority of soil lipids, their identity is currently unknown let alone their response to environmental change. A few key microbial lipids have provided insights into C cycling and climate variability [24], warranting a closer and more comprehensive understanding of what types of biogeochemical information can be harnessed from intact soil lipids.

For soil microorganisms, the main types of lipid analyses that have been widely studied to date are phospholipid fatty acids (PLFAs) and fatty acid methyl esters (FAME), which have provided a quantitative measure of bacterial and fungal biomass [25], a molecular signature of microbial guilds that are present in environmental samples [26–28], and a means to track the assimilation of C substrates [29]. However, both PLFA and FAME analyses profile fatty acids that have been cleaved from either phospholipids or from all ester-linked lipids, respectively. In doing so, these techniques fail to capture information on the intact lipid molecular structure from which the fatty acids originate. PLFA targets phospholipids, which represent only a fraction of the entire lipidome in soil and therefore omits other potentially interesting and informative lipid classes. The longstanding focus on glycerophospholipids as a proxy for membrane lipids has meant that little is known about other lipids common in soil microbes—such as glycerolipids and sphingolipids. This is a critical knowledge gap because, as this study demonstrates, these lipids are very responsive to environmental perturbation. For instance, during phosphorus limitation, which can be an indirect effect of drought, bacteria replace their membrane phospholipids with phosphate-free betaine lipids and even novel sphingolipids [30]. By contrast, the study of intact lipids via global or targeted lipidomics can provide deeper structural insights, enabling improved characterization of how microbial communities in natural settings adapt to environmental change [31–35].

Here we report a comprehensive lipidomics analysis of an arid grassland soil incubation experiment that simulates the simultaneous temperature and moisture stress of summer drought, followed by rewetting. This study addresses an important knowledge gap surrounding the rapid microbial response to wet up after a summer drought by evaluating the lipidome and metabolome during the 3-h period immediately after wetting. This is an important window of time during which a burst of respiration that is much higher than the basal level is commonly observed [8, 36–38]. We hypothesized that like respiration, distinct metabolic changes at the lipid level will rapidly occur in response to rewetting a dry soil. We expected to observe increased abundances of lipids associated with microbial stress tolerance and remodeling of cellular lipids due to reduced nutrient transport under the dry condition. We also hypothesized that the lipidome would be altered upon rewetting, reflecting an adaptive response when the water deficit is relieved. We demonstrate the immense potential for harnessing information from the soil lipidome to understand how soil microbiomes adapt and respond to stress. Comprehensive coverage of a broad range of lipid classes combined with our ability to characterize the fatty acid compositions of the measured lipids is an important advantage of our work, enabling the discovery of previously unknown responses at the lipid subclass and fatty acid level. Our findings suggest that lipids may be critical in orchestrating the broad differences in stress response strategies used by bacteria and fungi to survive environmental stress. Furthermore, our findings demonstrate the value of investigating the soil lipidome as a direct measure of the soil community’s physiological response to perturbation.

## Methods

### Field sampling and laboratory incubations

The soil was collected in October 2017 from the Washington State University Irrigated Agriculture Research and Extension Center field site located in Prosser, WA, USA (46° 15′ 04′′ N and 119° 43′ 43′′ W). The site and soil characteristics were described previously [39]. The soil represents a Warden silt loam that is characterized as a coarse-silty, mixed, super active, and mesic Xeric Haplocambid. The soil represents a marginal soil with low organic matter content (3.7%), low water holding capacity (24%), and pH of 8. It is not uncommon for summertime high temperatures to exceed 40°C in the semi-arid desert climate of Eastern Washington, where our field site is located, and such conditions are becoming more prevalent in many regions due to climate change [40–42]. The hot summers in this region are also the driest time of the year with under 5 mm of average precipitation in July. Samples from this grassland were homogenized using a 4-mm sieve and stored at 4°C, then incubated at 45°C for 7 days to simulate a summer drought. The soil was determined to be completely dry when no difference in weight was observed over a 24-h period. Aliquots of dried soil (15 g) were weighed into 50-mL Olympus tubes for each incubation sample (Genesee Scientific Corporation, San Diego, CA). Five independent replicates were established for each of the 6 sampling time points (0, 10, 20, 30, 90, or 180 min), resulting in a total of 30 incubation chambers. After harvesting the 0 min samples, the remaining 24 replicates were wet to 19% gravimetric water content using sterile DI water and incubated at 36°C for 10, 20, 30, 90, or 180 min. Nineteen percent gravimetric water was chosen to realistically mimic a precipitation event relevant to our field conditions, given the low water holding capacity of the soil and low average summertime precipitation. At each time point, 5 replicates were flash-frozen in liquid N and stored at −80°C.

### Sample extraction for metabolomics and lipidomics

A modified Folch extraction, MPLEx [43, 44], was used for the extraction of polar metabolites and lipids from soil samples. Briefly, 10 mL of stainless steel and garnet beads were added to each soil sample, followed by the addition of 20 mL of cold 2:1 (v/v) mixture of chloroform: methanol. Samples were then vortexed into a solution for 30 s, and 4 mL of cold Milli-Q water was added to each tube. Samples were horizontally vortexed for 10 min at 4°C and then allowed to completely cool. Each sample was probe sonicated with a 6-mm (1/4”) probe at amplitude 60% for 1 min each and then cooled. This was followed by horizontal vortexing for 2 h, cooling at −80°C for 15 min, probe sonication for 1 min, and cooling at −80°C. Samples were centrifuged at 4000 g for 5 min at 4°C resulting in separation into three defined upper layers: the upper-most aqueous (metabolite) layer, the protein interlayer, and the lower organic (lipid) layer with the remaining debris at the bottom. The polar metabolite and lipid fractions were collected separately in 20-mL glass vials and dried in a vacuum concentrator (Labconco, Kansas City, MO). Dried extracts were reconstituted in 1 mL of 2:1 chloroform to methanol and transferred to 1.7 mL SafeSeal tubes (Sorenson Bioscience Inc., Salt Lake City, UT), centrifuged at 9000 g for 5 min at 4° C to remove any debris, and the supernatant was transferred to 1.6 mL total recovery autosampler vials (Waters, Milford, MA). Samples were stored at −20°C until further analysis. Dried metabolite extracts were derivatized (see Supplementary Info for detailed methods) prior to gas-chromatography mass spectrometry (GC-MS) analysis.

### Mass spectrometry and data analysis

To characterize changes in the intact lipid profile in response to drying and rewetting, the total lipid extracts (TLEs) were analyzed by reversed-phase LC-ESI-MS/MS using a Waters Aquity UPLC H class system (Waters Corp., Milford, MA) coupled with a Velos Pro Orbitrap mass spectrometer (Thermo Scientific, San Jose, CA). TLEs were stored in 2:1 chloroform to methanol and evaporated then reconstituted at 9:1 chloroform to methanol mixture prior to injecting 10 μl onto a Waters column (CSH 3.0 mm × 150 mm × 1.7-μm particle size) maintained at 42°C. Lipid species were separated using a 34-min gradient elution at a flow rate of 250 μl/min. Mobile phases A and B consisted of ACN/H_2_O (40:60) containing 10 mM ammonium acetate and ACN/IPA (10:90) containing 10 mM ammonium acetate, respectively. The full gradient profile was as follows (min, %B): 0,40; 2,50; 3,60; 12,70; 15,75; 17,78; 19,85; 22,92 25,99; 34,99; and 34.5,40. The UPLC system used a Thermo HESI source coupled to the mass spectrometer inlet. The MS inlet and HESI source were both maintained at 350°C with a spray voltage of 3.5kV and sheath, auxiliary, and sweep gas flows of 45, 30, and 2, respectively. Each TLE was analyzed in both positive and negative ion modes in separate runs. Lipids were fragmented by both HCD (higher-energy collision dissociation) and CID (collision-induced dissociation) using a precursor scan of *m/z* 200–2000 at a mass resolution of 60k followed by data-dependent MS/MS of the top 4 ions. An isolation width of 2 *m/z* units and a maximum charge state of 2 were used for both CID and HCD scans. Normalized collision energies for CID and HCD were 35 and 30, respectively. CID spectra were acquired in the ion trap using an activation *Q* value of 0.18, while HCD spectra were acquired in the Orbitrap at a mass resolution of 7.5k and a first fixed mass of *m/z* 90. Confident lipid identifications (Supplementary Table S1) were made using LIQUID [45], and the tandem mass spectra and corresponding fragment ions, mass measurement error, and retention time were manually examined. LIQUID allowed the identification of co-eluting species and the presence of structural isomers that were separated using reverse-phase LC. For the relative quantification of lipids, a reference database containing identified lipids and observed *m/z*, and retention time was used for the alignment of lipid features using MZmine 2 [46]. Aligned features were manually verified, and peak apex intensities were used for downstream statistical analysis for the identified lipids. Positive and negative mode data were analyzed separately. Enrichment analysis of lipid ontology terms was done using the Lipid Mini-on [47] online tool based on a Fisher’s exact test (*p*<0.05). Details of the untargeted analysis of polar metabolites using GC-MS are provided in the Supplementary Info.

### Statistics and lipid ontology enrichment analysis

Statistical analysis of metabolomics and lipidomics data was performed using the pmartR package [48] with R version 4.0.2 [49]. Data were log2-transformed and normalized via global median centering. Statistical comparisons were performed for each time point after soil rewetting (10, 20, 30, 90,180 min) back to the dry 0-min soil group using ANOVA with a Dunnett test correction [50, 51]. For each lipid and polar metabolite, these adjusted *p* values and the mean log2 fold changes for each of the above comparisons are reported (Supplementary Tables S2 and S4), along with the number of observations per group. Lipid ontology enrichment [47] was used to extract biological information from the lipid name, highlighting significant trends in lipid categories, lipid class, sub-class, fatty acyl chain characteristics, total number of fatty acid carbons, and double bonds (Supplementary Table S3). Enrichment analysis was done separately for subsets of lipids that were significantly more abundant in either dry soil or wet soil to uncover significant shifts with change in soil moisture.

### Amplicon sequencing and data analysis

DNA was extracted from the soil samples using the Qiagen PowerSoil® DNA Isolation Kit (Qiagen, Germantown MD). Amplicon analysis was carried out as described previously [39] with 16S rRNA gene primers targeting the V4 hypervariable region of the 16S small-subunit (SSU) [52]. The ITS primers used were the ITS1f and ITS2 primers targeting the ITS1 region [53]. Amplicons were sequenced on an Illumina MiSeq using the 500-cycle MiSeq Reagent Kit v2 (Illumina, San Diego CA) according to the manufacturer’s instructions. QIIME2 (v2021.4) was used to denoise resulting Illumina MiSeq reads via DADA2 (*q2-dada2*), cluster amplicon sequence variants (ASVs), and assign taxonomy (*q2-feature-classifer*) using the SILVA database (v138) for 16S rRNA gene amplicons and the UNITE database (v8-10.05.2021) for ITS amplicons [54–57]. Statistical analyses on 16S rRNA gene and ITS datasets were performed using the program R, with the ggplot2, ggpubr, and gridExtra packages used to generate figures [49, 58–60]. First, poorly sequenced samples (<10,000 total counts) were removed from the dataset. The ASV datasets were rarefied (*rrarefy*; vegan package v2.5-7 [61]) based on the sample with the lowest ASV count (11,027 counts for the 16S rRNA gene dataset and 12,408 counts for the ITS dataset). Multivariate differences were detected by performing a Bray-Curtis dissimilarity-based (*vegdist*; vegan package v2.5-7) principal coordinate analysis (PCoA; *pcoa*, ape package v5 [62]). PERMANOVA was conducted using the “adonis” function inside the “vegan” package (Supplementary Table S5). Differences in phylum relative abundance through time were measured using an ANOVA, with pairwise time differences within phyla conducted using the Student’s *t* test and a false-discovery rate *p* value adjustment (Supplementary Tables S6 and S7). A differential abundance analysis on the rarefied data was conducted using DESeq2 in order to detect ASVs which were significantly more abundant at the 180-min time point (wet) as compared initial time point (dry) [63]. In order to assist in visualization, results were plotted at the genus level. Feature volatility analysis was conducted using a q2-longitudinal plugin [64] in QIIME2 in order to identify important features or ASVs (importance > 1%) with temporal dynamics most predictive of the sample state over time and visualize the longitudinal abundance (volatility plots) of these features. In this analysis, random forest machine learning regressors are used to identify “important” features (including low-abundance features) that change over time, and whose abundance is predictive of the specific time point, indicating a temporal relationship. Note that feature importance is intended as an exploratory method for identifying potentially relevant features for subsequent investigation and does not indicate statistical significance [64].

### Correlation network analysis

Amplicon (rarefied ASV 16S rRNA gene and ITS region sequence counts) and lipidomics data (normalized relative abundance) from the same samples, across all 6 timepoints (0, 10, 20, 30, 90, and 180 min) were matched and concatenated as a data matrix to perform a Pearson correlation using the R “cor.test” command. A correlation threshold of 0.75 and a *p* value threshold of 0.05 was used and rows with greater than 75% missing data were removed. Hierarchical All-against-All Association testing (HAllA) [65] was also applied to the same data (similarity measure: Pearson correlation, FDR correction: Benjamini–Hochberg–Yekutieli) to control the false discovery rate (FDR). The resulting network was visualized using Cytoscape [66].

## Results

### Characterization of the soil lipidome

To identify microbial traits associated with summer drought and rewet events, we investigated the short-term, rapid physiological response of an arid grassland microbiome to a simulated drought-rewetting event. Samples were collected following a drying period of 7 days (0 min, dry soil) and then at 10, 20, 30, 90, and 180 min after rewetting. Lipid and metabolite data were collected from all of the samples to determine the response of the soil microbiome to the different conditions. Lipid classes that were most prevalent under drought with elevated temperatures, and most sensitive to rewetting events, were identified by an untargeted approach that provided broad coverage of the soil lipidome. A total of 837 unique lipids were identified in positive and negative ionization modes, from the soil samples across all 6 timepoints.

**Fig. 1:**
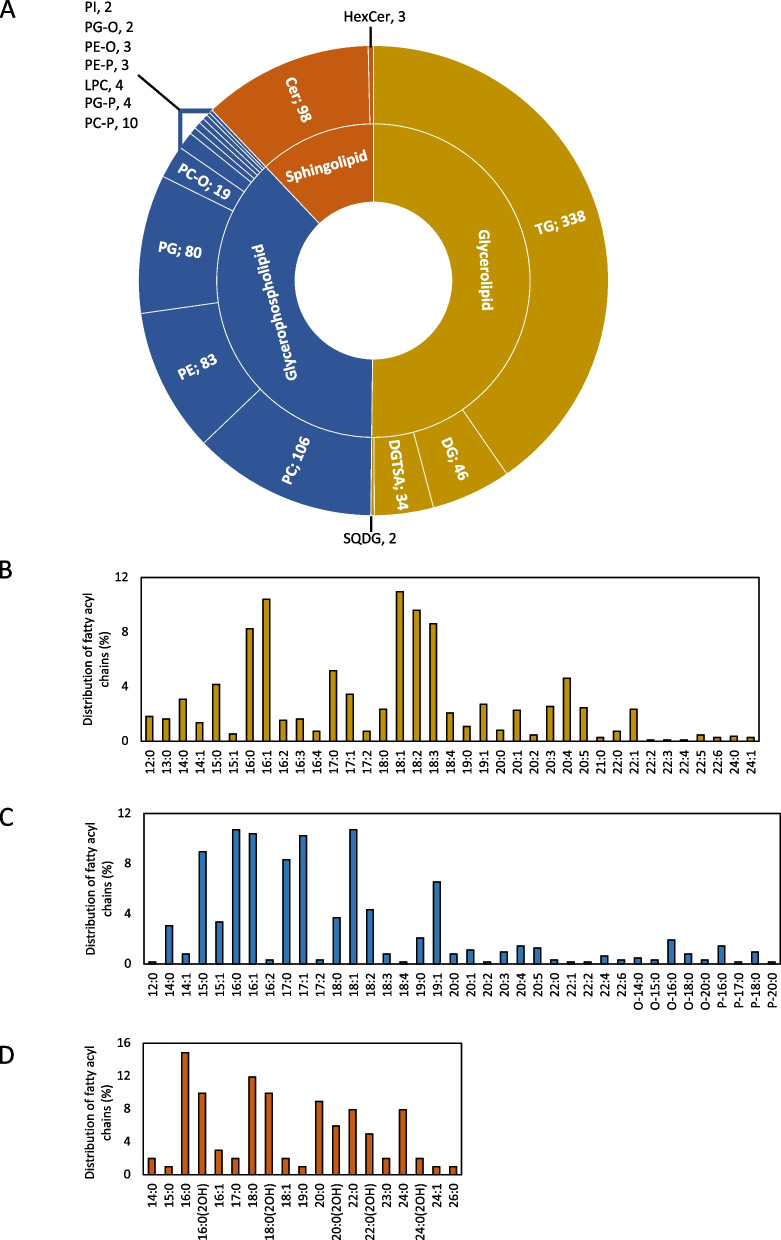
Distribution of lipids identified in soil samples from all time points. **A** Lipids were identified across 3 lipid categories [glycerolipids (yellow) glycerophospholipids (blue) and sphingolipids (orange)] and 18 sub-classes. Major sub-classes with greater than 10 lipids are shown. PC diacylglycerophosphocholine, LPC monoacylglycerophosphocholine, PE diacylglycerophosphoethanolmine, PG diacylglycerophosphoglycerol, PX-O ether PX, PX-P plasmalogen PX, DG diacylglyceride, TG triacylglyceride, DGTSA betaine lipid, and Cer ceramide. The number of lipids identified is indicated next to the subclass. Distribution of fatty acyl chains in **B** glycerolipid, **C** glycerophospholipid, and **D** sphingolipid categories

We characterized lipids across three lipid categories—glycerolipids, glycerophospholipids, and sphingolipids–and 18 lipid subclasses (Fig. 1A and Supplementary Table S1). Fifty percent (420) of unique lipids identified belonged to the glycerolipid category. These included species belonging to subclasses diacylglyceride (DG), sulfoquinovosyl diacylglycerol (SQDG), betaine lipids (DGTSA), and triacylglyceride (TG). Here, DGTSA represents two types of betaine lipids—1,2-diacylglyceryl-3-O-4’-(N,N,N-trimethyl)-homoserine (DGTS) and 1,2-diacylglyceryl-3-O-2’-(hydroxymethyl)-(N,N,N-trimethyl)-β-alanine (DGTA). The majority of the glycerolipids identified, 338 of the 420 species, belonged to the TG subclass. We also identified 319 (38%) unique lipids in the glycerophospholipid category. Those that we identified belonged to multiple subclasses (Fig. 1A), including diacylglycerophosphocholine (PC), diacylglycerophosphoethanolamine (PE), diacylglycerophosphoglycerol (PG), and diacylglycerophosphoinositol (PI). Ether-phospholipids characterized by an alkyl (PC-O, PE-O, and PG-O) or 1Z-alkenyl ether (PC-P, PE-P, and PG-P, known as plasmalogens) substituent at the sn-1 position of the glycerol backbone were also identified. Finally, we identified 101 (12%) sphingolipids, including ceramides (Cerd), phytoceramides (Cert), and hexosylceramides (HexCer). A significant strength of our study over previous untargeted lipidomics studies in the soil is that we were able to determine the chain length and degree of unsaturation of the individual fatty acid chains in each lipid. Using this approach, the percent distribution of fatty acyl chains in unique lipid species was identified across the three lipid categories (Fig. 1B–D). We observed the presence of a wide range of fatty acyl chain lengths (12 to 24 C) and determined their degree of unsaturation (0 to 6) across both the glycerolipid and glycerophospholipid categories. Relatively fewer shorter-chain fatty acyl chains were present amongst the sphingolipids which ranged between 14 to 26 carbons in length and were either saturated or monounsaturated. 2’-Hydoxylation of the fatty acyl chain was observed in specific fatty acids among the sphingolipids.

### Unknown features in the soil lipidome

Of the 1653 LC-MS features with MS/MS spectra, only 470 (28.4%) were annotated. Therefore, the majority of the soil lipidome remains uncharacterized, which is the current bottleneck of metabolomics due to challenges associated with accurate compound identification. A large number of metabolites or lipids features detected during untargeted profiling remain unknown and majority of studies typically report less than 20% identification. Although we mainly focus on the identified subset of the lipidome, we examined how the total lipidome, including unknowns, might be changed following the rewetting of dry soil, across the time points in the study (See Supplementary information).

### Lipidome shifts with changes in soil moisture and temperature

**Fig. 2:**
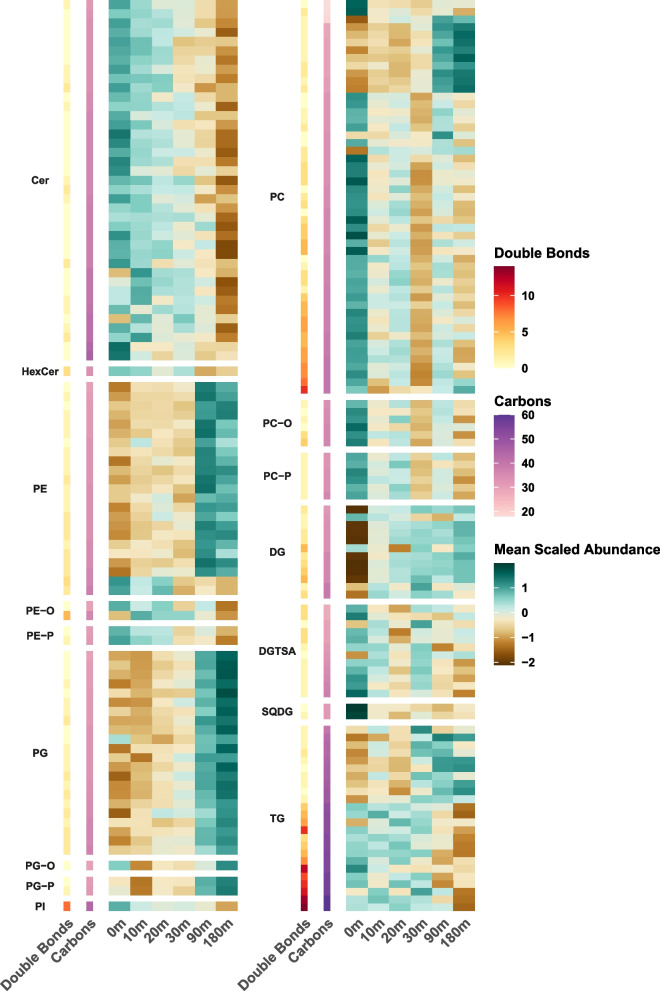
Heatmaps of mean auto-scaled log2 normalized abundance for lipid species that differed significantly between a later time point following soil wetting and the initial time 0 for dry soil (Dunnett-adjusted *p* value < 0.05 for at least one comparison). Heatmaps contain lipids from both positive and negative ionization modes, where each lipid species was auto-scaled individually; the mean for each time point is displayed in the heatmap. Sum total fatty acid carbons and double bonds are indicated

Relative quantification of identified lipids across all samples, before and within 180 min after rewetting, revealed significant changes in the soil lipidome. Statistical comparisons to the dry (0 min) soil group were performed for each time point after soil wetting (10, 20, 30, 90,180 min). Nearly half of the lipid species, 43.8% (206 out of 470), significantly changed (Dunnet-corrected *p* value<0.05) in abundance in at least one of the pair-wise comparisons during the 180 min window of time following wetting. The mean log2 fold change for these lipids ranged from −2.16 to 2.55 (Fig. 2, Supplementary Table S2). While some lipids (46 out of 206; 22%) showed significant changes in abundance as early as 10 min following rewetting, the majority of the lipid species (160 out of 206; 78%) were significantly different by the end of the incubation (0 min versus 180 min). The main findings for each lipid category are detailed in the following sections.

### Glycerolipids

The majority of the lipid species identified in the soil samples belonged to the glycerolipid category, and 22% (50 out of 230) of these had statistically significant changes in abundance following rewetting. Of the DGTSA, 83% (10 out of 12) were significantly more abundant in dry soil, prior to rewetting. The two lipid species that were exceptions were DGTSA (15:0/15:0) and DGTSA (15:0/18:0), which showed the opposite trend. Interestingly, these two were the only DGTSA lipids with no double bonds (saturated) and both contained the 15:0 fatty acid suggestive of bacterial origin. Only two SQDG lipids were identified, and these were also significantly more abundant in dry soil. Both DGTSA and SQDG are phosphorus-free membrane lipids.

Most of the significantly shifting DG lipids (10 out of 12) showed increased abundances in response to soil rewetting. For the TG lipids that showed significant changes in abundance with soil rewetting, the direction of change depended on the sum of total fatty acid carbons and the sum of double bonds. Within the significantly changing TG lipids, 41.6% (10 out of 24) increased in abundance after soil rewetting and contained between 40 and 48 total fatty acid Cs and 0–2 double bonds. These TG lipids comprised fatty acids with C chain lengths between 12 and 18 that were either saturated or monounsaturated. By contrast, 50% (12 out of 24) of TG lipids, having longer fatty acid chain lengths and polyunsaturation, were significantly more abundant in dry soil. These lipids contained longer sum total fatty acid C, ranging from 51 to 60 C with 3–14 total double bonds. The remaining two TG lipids showed no significant change in abundance following rewetting.

### Glycerophospholipids

A majority, 63.3% (116 out of 183) of the detected glycerophospholipids significantly changed in abundance following soil rewetting. The bulk of responsive lipids in subclass PE (21 out of 23; 91.3%) increased after rewetting. These PE lipids contained between 30 and 36 fatty acid C and up to 2 double bonds. Only two PE lipids were significantly more abundant in the dry soil and had either a higher sum total of fatty acid C (38) or total number of double bonds (3). Thus, the direction of change trended with the sum of the total fatty acid C and double bonds, similar to what was observed for the TG lipids. All the PE lipids comprised fatty acids with chain lengths between 14 and 20 C and 0–2 double bonds. Similar to the PE lipids, all the responsive PG lipids significantly increased in abundance after rewetting. The trend of PC, PC-O, and PC-P lipids depended on the sum total fatty acid C. Of the significant PC lipids, 20.8% (10 out of 48) were higher in abundance after rewet and had between 19 and 31 total fatty acid C and up to 2 total double bonds. Most of these PC lipid species contained 14:0, 14:1, 15:0, or 15:1 fatty acids. In dry soil, 77 % (37 out of 48) of the significant PC lipids and all of the significant PC-P and PC-O lipid species were higher in abundance and they had between 33 and 40 total fatty acid C and up to 10 double bonds.

### Sphingolipids

For the identified sphingolipid species, 70.2% (40 out of 57) had statistically significant changes upon rewetting. Of these, 95% (38 out of 40) were higher in abundance in dry soils with a decreasing trend after rewetting (Cer and HexCer in Fig. 2).

An enrichment analysis of lipid ontology terms (Supplementary Table S3) indicated that sphingolipids were enriched in the subset of lipids that were significantly higher in dry soils. Polyunsaturated glycerophospholipids, such as those with polyunsaturated fatty acid (PUFA) chains 18:2, 18:3, 20:3, 20:4, 20:5, and glycerolipids with PUFA chains 18:2, 18:4 were also enriched in dry soil. An enrichment in glycerophospholipids with 3, 5, and 6 double bonds and in triacylglycerols with 4 and 10 double bonds was observed in dry soil. In contrast, saturated and monounsaturated triacylglycerols and glycerophospholipids were enriched in the subset of lipids with higher abundance after soil rewetting. The lipids that were more abundant in wet soils included glycerophospholipids (PG, PE). In addition, saturated and monounsaturated fatty acid chains were enriched in triacylglycerols (14:0, 14:1 15:0, 16:0) in wet soil. The relative percent distribution of the fatty acid chains present in intact lipids (glycerolipids, glycerophospholipids, and sphingolipids respectively) that were significantly more abundant in either dry (0 min) or wet (10-180 min) soils are shown in Supplementary Fig. S1.

### Characterization of the polar metabolome

**Fig. 3:**
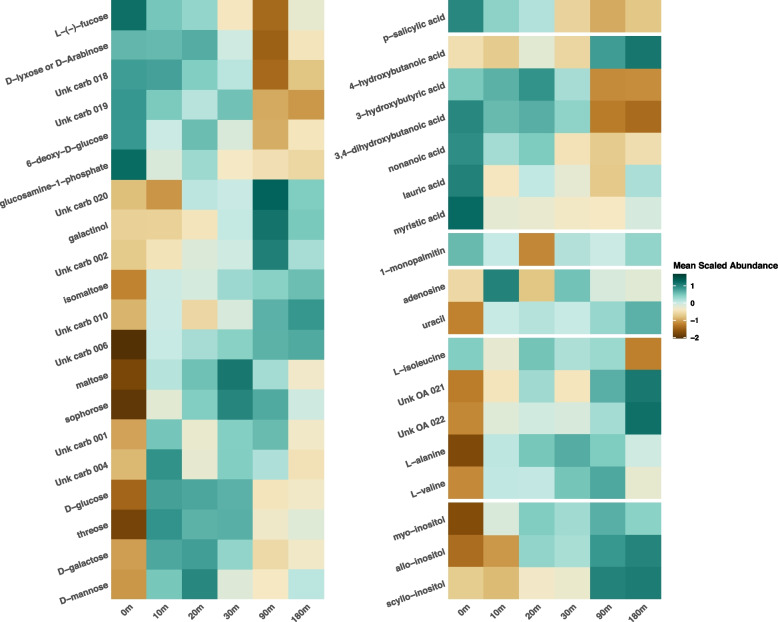
Heatmaps of mean auto-scaled log2 normalized abundance for polar metabolites that differed significantly between a later time point following soil wetting and the initial time 0 for dry soil (Dunnett-adjusted *p* value < 0.05 for at least one comparison). Each metabolite was auto-scaled individually, and the mean for each time point is displayed in the heatmap

Untargeted metabolomics analysis using gas chromatography coupled mass spectrometry (GC-MS) detected 244 features, of which 52 were identified and 22 were putatively identified by superclass across eight superclasses. The majority of the identified metabolites were carbohydrates (46 identifications comprising monosaccharides, disaccharides, sugar alcohols, oligosaccharides, and glycosyl compounds) and fatty acyls (14 identifications comprising hydroxy fatty acids, saturated fatty acids, and fatty nitriles). Relative quantification of polar metabolites across all samples indicated that the soil metabolome shifted in composition following rewetting. Statistical comparisons to the dry, 0-min soil group, were performed for each time point after soil wetting (10, 20, 30, 90, 180 min), and a total of 39 identified metabolites showed statistically significant changes (Fig. 3, Supplementary Table S4) in abundance across all the comparisons. At the superclass level, these metabolites were comprised of carbohydrates (21), fatty acyls (6), organic acids (5), organic oxygen compounds (3), nucleic acids (2), a glycerolipid, and a benzenoid. Amongst the carbohydrates, while no clear trend in the direction of change was apparent in the monosaccharides, the disaccharide metabolites were less abundant in dry soil and increased in abundance after rewetting. Inositols, nucleic acids, organic acids, and 2 of the 3 amino acids also showed an increasing trend upon rewet. The majority (6 out of 7) of the fatty acid and lipid-related metabolites were significantly more abundant in dry soil.

### Bacterial and fungal community response to soil rewetting

**Fig. 4:**
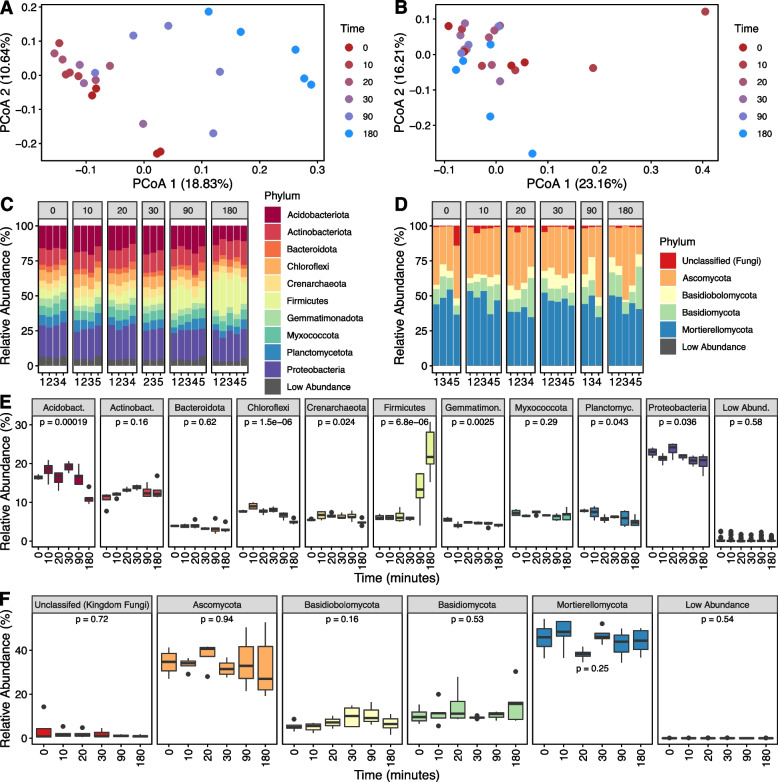
Analysis of archaeal/bacterial and fungal community composition following 180 min of rewetting of dessicated soil. PCoA of **A** 16S rRNA gene (archaeal/bacterial) and **B** ITS region (fungal) amplicon sequencing datasets. Stacked bar charts illustrate the relative abundance of the archaeal/bacterial (**C**) and fungal (**D**) taxa which comprise at least 5% of the respective microbial community; phyla which are <5% are considered “low abundance.” A comparative analysis through time is presented as boxplots in panels (**E**) 16S rRNA gene (archaeal/bacterial) and **F** ITS region (fungal). *P* values represent the results of an ANOVA with values < 0.05 indicating that a significant difference in relative abundance exists between at least two timepoints. Pairwise *t* test comparisons through time and corresponding *p* values can be found in Supplemental Tables S6 and S7

The microbial community response over time to soil drying and rewetting was analyzed by amplicon sequencing of the 16S rRNA gene for bacterial/archaeal and ITS region for fungal communities. Multivariate analyses via principal coordinate analyses and associated PERMANOVA tests revealed that time significantly impacted both the bacterial/archaeal (*p* = 0.001; Fig. 4A) and the fungal community structures (*p* = 0.006; Fig. 4B). Statistically significant *p* values (*p* < 0.05) were observed in the pairwise PERMANOVA analyses (Supplementary Table S5) for the bacterial/archaeal community as early as 10 min after rewetting. Significant *p* values were also obtained for 0 vs 20 min, 0 vs 90 min, and 0 vs 180 min pairwise comparisons but not for the 0 vs 30 min comparison. In contrast, for the fungal community data, the only significant pairwise comparisons were 0 vs 180 min, 10 vs 180 min, 20 vs 180 min, and 30 vs 180 min indicating no detectable shifts in the fungal community until 180 min.

When analyzing the microbial community members that were driving these multivariate differences, we observed that the bacterial/archaeal community was, on average, dominated by the phyla Proteobacteria (21.7%), Acidobacteriota (16 %), Actinobacteriota (12.6%), and Firmicutes (10.9%); all other taxa were <10% on average (Fig. 4C). Meanwhile, fungal communities were dominated by Mortierellomycota (44.4%), followed by Ascomycota (34%) and Basidiomycota (12.1%), while all other fungal taxa were lower in abundance (Fig. 4D). After rewetting, there was a significant increase in the relative abundance in members of the Firmicutes phylum (ANOVA *p* value <0.001) and a significant decrease in members of the phyla Acidobacteriota (*p* value <0.001), Chloroflexi (*p* value <0.001), Crenarchaeota (*p* value 0.024), Gemmtimonadota (*p* value 0.003), Planctomycetota (*p* value: 0.043), and Proteobacteria (*p* value: 0.036) (Fig. 4E, Supplementary Table S6). By contrast, there were no significant shifts in the relative abundances of fungal phyla following rewetting (Fig. 4F, Supplementary Table S7).

**Fig. 5:**
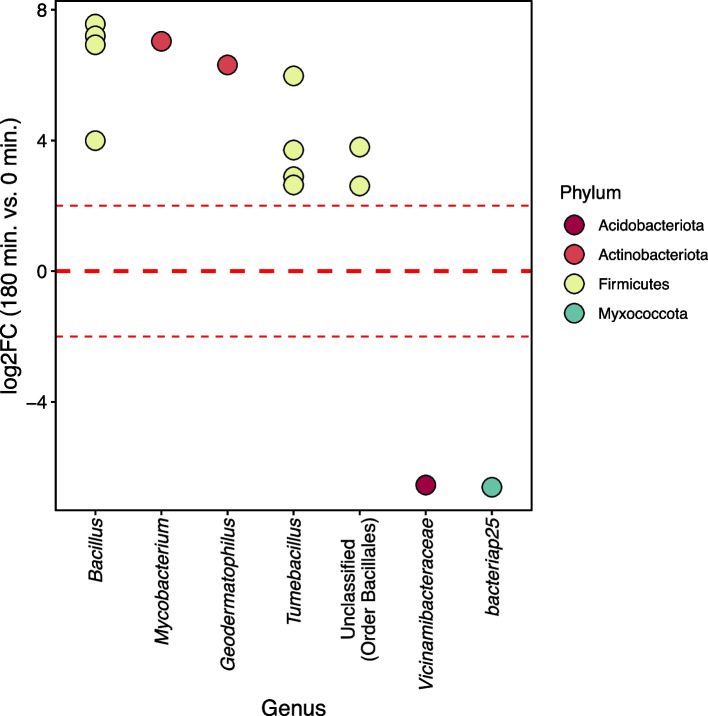
Differentially abundant ASVs for the archaeal/bacterial datasets across the two extreme timepoints: 0 min (dry) and 180 min (wet). The points represent the log2 fold change of an ASV belonging to the genus outlined on the *x*-axis, colored based upon the corresponding phylum

DESeq2 was used to identify differentially abundant ASVs between the most divergent timepoints 0 min (dry) and 180 min (3h after rewet) (Fig. 5) to better understand the response of the bacterial community to soil drying and rewetting conditions. There were 12 bacterial ASVs that were significantly more abundant after the 180 min incubation (Fig. 5). Most of these ASVs (10) belonged to the Firmicutes and were distributed across three genera: *Bacillus*, *Tumebacillus*, and an unknown genus from the order Bacillales. In order to put these differences in context, the average relative abundances before DESeq2 normalization for these taxa at 180 min ranged from 0.11 to 6.63%. The largest increases in taxa from 0 min to 180 min were for a *Tumebacillus* ASV (from 0.93 to 5.7% relative abundance) and an order Bacilliales ASV (from 1.21 to 6.63% relative abundance). The remaining two ASVs were Actinobacteria from the genera *Mycobacterium* and *Geodermatophilus*. There was only a single ASV belonging to the genus *Clarireedia* that was significantly more abundant within the fungal community after 180 min, likely due to it being below detection at the 0 time point (average rarefied counts at 0 minutes: 0; average rarefied counts at 180 minutes: 1285). Comparisons between 180 min or 0 min and 10, 20, 30, or 90 min timepoints are shown in Supplementary Figs. S8 and S9.

**Fig. 6:**
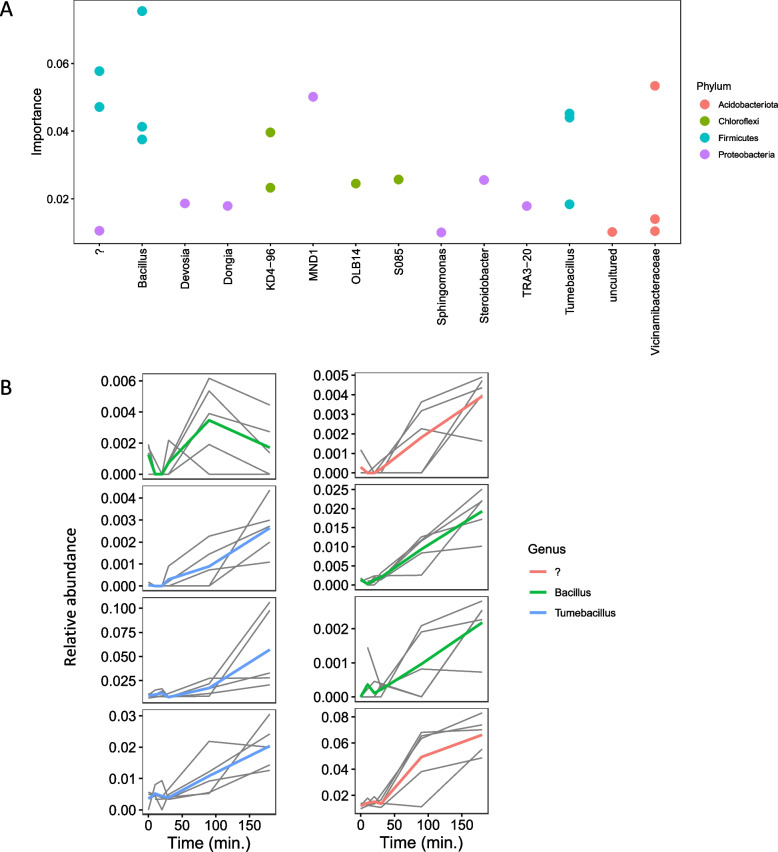
Longitudinal feature-volatility analysis of bacterial/archaeal ASVs. **A** The points represent the importance of an ASV belonging to the genus outlined on the *x*-axis, colored based on the corresponding phylum. Only ASVs with an importance > 1% are shown. **B** Relative abundances of the ASVs belonging to the Firmicutes phylum are shown across time for individual replicates (narrow black lines) and for group averages with thick lines colored based on genus

To better understand the volatility or variance of the relative abundance of bacterial/archaeal taxa over time, feature volatility analysis was used to identify the bacterial taxa that were most important in capturing the temporal dynamics in this experiment. Twenty-three bacterial ASVs had an importance > 1% (Fig. 6A), and the highest number (8) of these belonged to Firmicutes. Figure 6B shows the longitudinal abundances of these 8 ASVs as volatility plots. The majority of these ASVs show an increasing trend in relative abundance after the 30-min timepoint following soil wetting. Six of these eight Firmicutes ASVs were also identified as differentially abundant in the DESeq2 analysis (Fig. 5) indicating an overlap in the findings from the two analyses.

### Integrative correlation network analysis

**Fig. 7:**
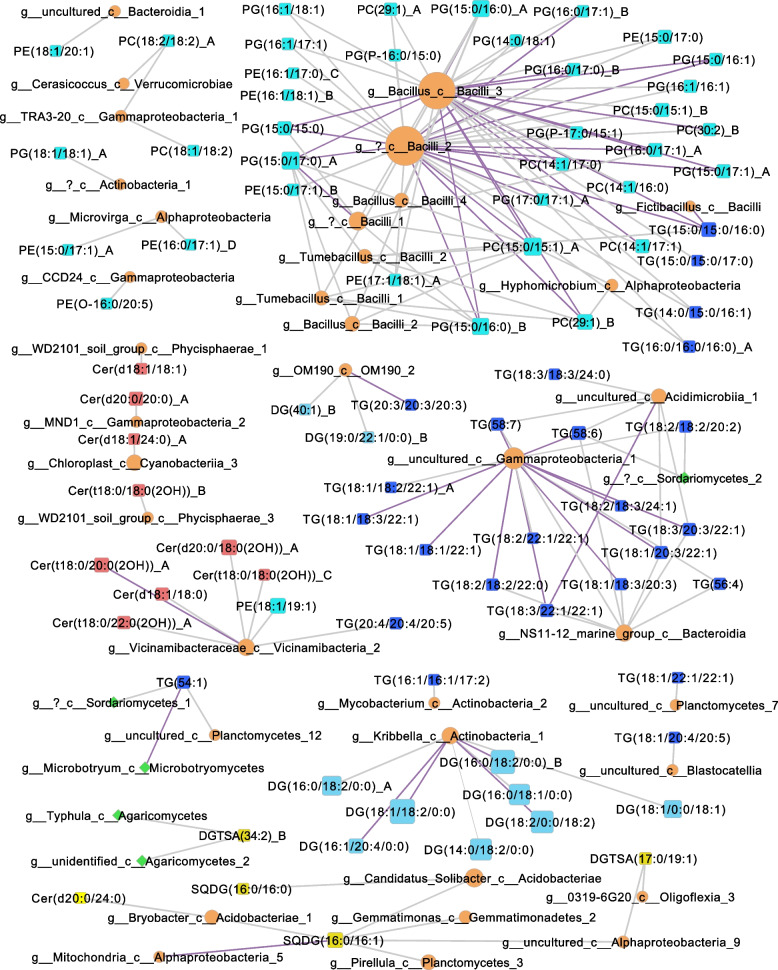
A network visualization of significant positive Pearson correlations between normalized lipid (squares) relative abundance and ASV counts from rarefied 16S rRNA gene (orange circles) and ITS region (green diamonds) amplicon data across all time points. Edge thickness is proportional to the correlation coefficient and node size is proportional to the number of connected edges. Edges are colored purple if FDR < 0.05. Bacterial and fungal nodes are labeled to indicate genus if available and class. Lipid node colors: glycerophospholipids, cyan; TGs, dark blue; DGs, light blue; and phosphorus-free betaine and SQDG lipids, yellow

Pearson correlations were calculated between lipid species and 16S rRNA gene/ITS region measurements across all time points to identify possible links between the soil microbiome and lipids (Supplementary Fig. S10, Supplementary Tables S8 and S9). One hundred sixty-four significant positive correlations (Fig. 7) were found between the microbiome and lipidome data pointing to potential relationships between specific microbial taxa and lipid species. Twenty-nine glycerophospholipids consisting of PC, PE, PG, and PG-P species, all of which significantly increased in abundance after soil rewetting, were correlated with at least one of the 8 ASVs corresponding to the Firmicutes phylum (class Bacilli).

These glycerophospholipids were all comprised of total fatty acid C ranging between 29 and 35 with up to two total double bonds. Two fungal ASVs belonging to class Agaricomycetes (genus *Typhula* and an unidentified genus) were correlated with the betaine lipid DGTSA (34:2) and two bacterial ASVs, belonging to the classes Alphaproteobacteria and Oligoflexia, were correlated with DGTSA (17:0/19:1). Five sphingolipids including ceramides and phytoceramides with 2’-hydroxylation of the fatty acid chain were correlated with an ASV belonging to the genus *Vicinamibacteraceae* and phylum Acidobacteriota and these lipids were significantly higher in abundance in dry soils. Eight DG lipids were correlated with an ASV belonging to the phylum Actinobacteriota and genus *Kribbella*. Two bacterial ASVs belonging to the phylum Actinobacteriota (genera *Mycobacterium* and uncultured) were correlated with one and six TG lipids respectively. DGs can serve as lipid precursors for TG biosynthesis and while TG occurrence is rare in bacteria, it is widespread among Actinobacteriota. An ASV of the class Gammaproteobacteria was correlated with 14 TG lipids. Three ASVs belonging to the phylum Planctomycetota were correlated with a total of 2 DG and 3 TG lipids.

## Discussion

Large rainfall events following drought are known to cause pulses of CO_2_, often many times greater than the basal level of soil respiration [37]. But the microbial drivers behind these respiration bursts remain elusive. As heatwaves and droughts are becoming more frequent and severe [1, 2], it is necessary to further reveal the physiological responses of soil microbial communities to drought-rewetting events. Our study uses a multi-omic approach to demonstrate distinctive physiological signatures of microbial communities in response to drying-rewetting events that can occur in many arid soils. Only a handful of studies [13, 35, 67–69] so far have investigated intact soil lipids, and this work represents the largest number of unique lipids identified in a soil microbiome to date.

### Nutrient limitation in dry soil induces replacement of membrane phospholipids

Our study demonstrated a strong lipidomic response to soil desiccation and rewetting. In particular, several bacteria remodeled their cell membrane lipids in response to contrasting resource access under dry and wet conditions. In dry soil, water is limited within soil pores in the soil matrix, which impedes the transport of C and nutrients and reduces nutrient access by microorganisms. The glycerophospholipids are the major structural components of lipid bilayers in all cell membranes and influence cellular adaptability to stress [18]. Under dry conditions, membrane glycerophospholipids such as PC, PE, and PG were depleted and replaced with membrane lipids that lack phosphorus, such as SQDG and DGTSA. Network analysis showed a significant correlation between DGTSA lipids and ASVs belonging to the Basidiomycota and Proteobacteria phyla and included species that are known to synthesize betaine lipids [70, 71]. Although DGTSA is not common in bacteria, its presence and accumulation have been seen previously in some Alphaproteobacteria under conditions of phosphate limitation [19, 72]. Replacing membrane phospholipids such as PC and PE with phosphorus-free membrane lipids can provide a fitness advantage by allowing microbes to conserve phosphate for other cellular processes [72–74] required during drought stress. Under phosphate-limiting conditions, existing membrane phospholipids can be degraded as a source of phosphorus for the synthesis of essential biomolecules [75]. After rewetting, phospholipid abundance rapidly increased, which is consistent with increased nutrient transport and access, and enhanced glycerophospholipid biosynthesis. These responses demonstrate a strong physiological response of the soil microbiome to rewetting.

The metabolomics data revealed that osmolyte accumulation was not the dominant physiological strategy employed by soil microorganisms in our soil under these experimental conditions. Although culture-based studies have shown microbial accumulation of compatible solutes under osmotic stress-induced using salt [76, 77], this has not been consistently observed in soil [11, 13, 37, 78]. Our results reveal that putative osmolytes including trehalose, mannitol, and glycerol did not change significantly in abundance after rewetting of dry soil. However, several metabolites, including some with known osmoprotective properties, such as amino acids, disaccharides, inositols, organic acids, and nucleic acids, increased in abundance after rewetting. This finding suggests that limited resource availability due to impeded diffusion in dry soils may affect microbial osmolyte production which would require substantial resources. An increase in the abundance of amino acids and nucleic acids upon rewetting of dry soil is likely due to the increased availability of nitrogen which is required for the synthesis of these compounds. We did, however, observe that glucosylglycerol, a compatible solute with osmoprotective properties was significantly more abundant in dry soil and showed a decreasing abundance over time upon rewetting. This suggests that certain microbes do produce osmoprotective metabolites during soil drying even if osmolyte production may not be a dominant acclimation strategy across the community.

### PUFAs and sphingolipids may provide stress tolerance in dry conditions

Our results suggest that specific lipids that are typically found in fungi and select bacteria may play a key role in the metabolic response and adaptation of these taxa to hot and dry soil conditions that are often experienced during summer droughts. In particular, PUFAs with longer fatty acyl chain lengths—18, 20 C—were more prevalent in the lipids in dry compared to wet soil. Specifically, glycerophospholipids and triacylglycerols with highly unsaturated fatty acyl chains, typical of eukaryotic organisms [79, 80], were more abundant in the drought-stressed soil. In fungi, fatty acid unsaturation and chain length are both known to play a role in mitigating the effects of osmotic and heat stress via a lipid-mediated downregulation of cellular stress response pathways [81, 82]. Nitrogen and phosphorus availability can also alter the fatty acid composition of lipids and nutrient-limited conditions, such as those in dry soil, can promote PUFA biosynthesis in microalgae and fungi [83, 84]. Similarly, sphingolipids (specifically ceramides) which are common in fungi, but less so in bacteria, increased in abundance following hot and dry incubation conditions. Sphingolipids are bioactive membrane lipids that have an important role in cell signaling and stress response [85, 86]. Elevated levels of these bioactive lipids are known to enhance heat and osmotic stress tolerance in yeast [87, 88]. Sphingolipids also regulate polarized hyphal growth in filamentous fungi [89] contributing to drought stress tolerance by increasing access to water and nutrients in dry soil.

Amongst the soil biota, while bacteria have distinctive fatty acid signatures, it is harder to discriminate between eukaryotes such as fungi, microalgae, plants, and protists [90]. Protists generally prefer moist environments and their abundances drastically reduce under extreme soil drying, although they can survive as cysts [91]. Fungi on the other hand are known to be more drought tolerant [3] which led us to associate eukaryotic-like lipids that were more abundant in dry soils with fungi. These included long-chain PUFAs such as 20:4 and 20:5, which are common in eukaryotes, but rare in bacteria. Additionally, the majority of the glycerophospholipids that were higher under hot and dry conditions were PCs, which are abundant and ubiquitous in membranes of eukaryotic cells. Although we make a general assumption in this study that the PUFAs are predominantly of fungal origin, it is important to note that it is possible that certain soil bacteria may also be capable of synthesizing these PUFAs. Long-chain PUFAs have been identified in select bacteria that inhibit high-pressure, low-temperature deep-sea habitats, and cold marine environments [92–94] and in terrestrial myxobacteria [95]. Given that our experimental incubations did not involve plants, we inferred that the lipids with eukaryotic characteristics are likely of fungal origin. However, they could also originate from soil protists, and it is not possible to exclude the possibility that plant lipids could persist in the soil.

Our results suggest that the biosynthesis of PC lipids, PUFAs, and sphingolipids may be a key metabolic response, as evidenced by elevated lipid signatures under drought conditions. The relationship between the ability of certain microbial taxa to synthesize these lipids and their tolerance to drought warrants further investigation. However, the physiological changes revealed in the lipidome were not accompanied by clear shifts in fungal community composition during our experiment. While some previous studies have shown that fungal community composition is less affected by drought [96, 97], other studies have observed a stronger functional response in the fungal community in comparison with bacteria [14]. Moreover, there was only a single fungal ASV belonging to the genus *Clarireedia* that was significantly more abundant at the 180-time point than the 0. This points to the likelihood of a predominantly physiological, rather than compositional response of soil fungi to drought stress in the first 180 min. This physiological response, as suggested by our results, has important implications for determining the community metaphenomic [98] response to environmental perturbations, that may not be detected by community profiling assays.

### Bacterial activity increases upon rewetting

Bacterial lipid metabolism and growth responded rapidly to rewetting. Lipids typically associated with bacteria [99], increased in abundance upon rewetting, including PG, PE, PC, and TG lipids with saturated or monounsaturated short fatty acid chains (14 and 15C). The increased abundance of membrane lipids (PG, PE, PC) and many primary metabolites (amino acids, nucleic acids, organic acids, sugars) within 180 min after rewetting suggests rapid growth [100]. Network analysis revealed significant correlations between many of these glycerophospholipid species and bacterial ASVs belonging to the phylum Firmicutes (class Bacilli), suggesting positive selection for Firmicutes during drought rewet events. Increases in the abundance of ASVs belonging to the phylum Firmicutes are consistent with the fitness advantages of thick cell walls that render gram-positive bacteria more resistant to stress associated with drying and rewetting. Firmicutes, and the genus *Bacillus* in particular, are known to form endospores, which aid their survival and resilience under adverse environmental conditions including desiccation stress [101]. The rapid growth of Firmicutes has previously been detected within 3 h of rewetting dry soil using quantitative stable isotope probing [15]. Although most studies typically measure microbial community composition days to months following rewet, the results from Blazewicz et al. [15] indicate that even within the first 3 h following wet-up, distinct population responses occurred in the community, underscoring the importance of the first few hours following rewet. In our study, 16S rRNA and ITS region amplicon sequencing reveal interesting dynamics, especially in certain members belonging to the phylum Firmicutes. Our analysis indicates that 6 ASVs belonging to the phylum Firmicutes not only changed significantly in relative abundance after soil wetting, but also had temporal trends that were predictive of the soil rewetting in the first 3 h. By contrast, the relative abundances of organisms known to thrive in arid environments, like Chloroflexi and Gemmatimonadota, decreased after rewetting [102]. Phyla with slow-growing members, such as Acidobacteriota and Planctomycetota [103–105], also diminished in relative abundance after rewetting. While these traits are beneficial in dry soil, fast growers dominate after rewetting events, when resource access is high [106]. The rapid change in water potential can also cause cell lysis and death upon rewetting [15]. The abovementioned bacterial glycerophospholipids and TGs with short, saturated, and monounsaturated fatty acids were less abundant in dry soil and increased over time upon rewetting implying that growth of Firmicutes and/or physiological responses from other bacterial phyla—even those that may not have experienced detectable changes in abundance—could be responsible for observed changes in the lipidome.

Correlations observed between multiple TG lipid species and Gammaproteobacteria were both unexpected and interesting, given that bacterial TG biosynthesis is thought to be largely restricted to the Actinomycetes phylum [107]. An alkane-degrading marine Gammaproteobacteria, *Alcanivorax borkumensis*, was found to synthesize TGs enriched in saturated fatty acids [108]. This is a compelling reason to further investigate TG biosynthesis in soil Gammaproteobacteria. Similarly, the correlations between ceramides and Acidobacteriota raise the question of whether members of this phylum produce sphingolipids. Sphingolipid production in bacteria is uncommon and based on current knowledge it is limited to members of Bacteriodetes, Chlorobi, and some Proteobacteria. However, it is possible and even likely that there exist yet unexplored soil bacteria that can synthesize sphingolipids and other lipids that are currently considered fungal [30]. Although correlation does not prove causation and statistically robust correlations between a lipid species and a microorganism may arise even from indirect interactions with other variables or features [109], the correlation analysis can help explore potential associations between taxa and lipids. Further investigation is warranted since the lipid composition of the vast majority of soil microorganisms has yet to be characterized.

### Perspectives and conclusions

Our research demonstrates the rich and largely untapped reservoir of lipids present in the soil microbiome and highlights the sensitivity of this pool for assaying physiological responses of microorganisms within the soil habitat. The wealth of dark matter within the lipidome may contain biochemical information critical to answering pressing soil ecology challenges, including predicting how microorganisms will respond to climate change. Future needs include the development of a taxonomic catalog of soil microbial lipids and a better understanding of how they change under varying environmental conditions. The development of a taxonomic catalog of soil microbial lipids and associated phenotypes could transform soil ecology by enabling an incredibly sensitive measure of both taxonomic and metabolic responses in a single assay. This would help determine if the ability to biosynthesize lipids with specific structural characteristics are functional traits that influence microbial fitness under environmental change across taxonomic groups. It is possible that soil environments may contain microbes with unique lipid compositions that defy what we currently consider to be bacterial or fungal. For instance, there may exist yet undiscovered bacteria, capable of biosynthesizing lipids that we currently assume are fungal, which makes them more resistant to drought stress. It is also likely that there exist several unknown lipid synthesis pathways. Our study employs conventional LC-MS/MS-based lipidomics which cannot ascertain the double bond position in the fatty acids and therefore significantly limits any taxonomic association of intact lipids to microbes. Systematic characterization of lipidomes of soil microbial isolates to characterize the relative distribution of intact lipids along with details of lipid structure, including double bond positions in fatty acids using multi-dimensional analytical platforms which incorporate ion mobility and ozone-induced dissociation (OzID) [110], can aid in more confident taxonomic associations. The ability to make high-throughput measurements of lipids in complex samples using OzID and automated data processing is a critical capability gap that needs to be addressed. Community-curated reference databases and advances in “standard-free” in silico approaches will further improve chemical space coverage and accurate identifications in metabolomics and lipidomics [111]. While this study uses amplicon sequencing of the 16S rRNA gene and ITS region for measuring the changes in the relative abundance of microbial taxa, quantitative stable isotope probing of approaches would be better suited to detect rapid population responses and measure taxon-specific growth rates [15]. Our data along with the findings of Blazewicz et al. [15] highlight the potential value of further investigating microbial population dynamics and metabolism during those initial minutes-hours which is currently under-studied.

Our study shows that the soil lipidome is a sensitive indicator of the soil microbial phenotypic response to abiotic stress, even at short time scales. Both lipidomic and metabolomic results point to physiological shifts in response to changes in nutrient access under drying-rewetting cycles. The simulated drought resulted in an increase in lipids implicated in mediating heat and osmotic stress and nutrient deprivation. It also induced elevated levels of lipids containing fatty acid moieties that were characteristic of fungal metabolism. This work reveals that functional traits such as polyunsaturated and longer fatty acid chain length and sphingolipid biosynthesis may regulate drought tolerance and affect microbial fitness during summer droughts. For the first time, we show evidence of elevated levels of ceramides in soil and increased prevalence of long-chain polyunsaturated fatty acids in glycerophospholipids and triacylglycerols following drying. The increase in lipids with fatty acids typical of bacteria following rewetting suggested rapid metabolic reactivation in the bacterial community as nutrient diffusion increased and conditions became more favorable for growth. These results suggest that different taxa may have distinct metabolic adaptation strategies when faced with water stress based on the composition of their lipidomes. Anticipated increases in drought due to climate change can differentially alter bacterial and fungal metabolism at the lipid level which can have important consequences on community-level functions. Our results demonstrate an exciting potential for lipidomics to provide function-driven in situ measurements of physiological responses and community-level phenotypes that occur over short time frames and may not be fully captured with genomic approaches alone.

## Supplementary Information

**Additional file 1: Supplementary Table S1.** Lipid class distribution; **Supplementary Table S2.** Lipidomics data statistical analysis; **Supplementary Table S3.** Lipid ontology enrichment; **Supplemental Table S4.** Metabolomics data statistical analysis; **Supplementary Table S5.** Bray-Curtis PERMANOVA statistical analyses; **Supplementary Table S6.** 16S rRNA gene amplicon sequencing pairwise t-tests; **Supplementary Table S7.** ITS region amplicon sequencing pairwise t-tests; **Supplementary Table S8.** Pearson correlation between lipid species and 16S rRNA gene/ITS region measurements; **Supplementary Table S9.** HAllA analysis.

**Additional file 2: Supplementary Figure S1.** Relative distribution of fatty acid chains; Unknown features in the soil lipidome; Statistical analysis on all lipid features (including unidentified); **Supplementary Figure S2.** Lipidomics PCA analysis; **Supplementary Figures S3 and S4.** Volcano plots for lipidomics positive and negative mode data; Classical molecular networking using GNPS; **Supplementary Figures S5-S7.** Molecular networks; **Supplementary Figures S8 and S9.** Differentially abundant ASVs in 16S rRNA and ITS region amplicon sequencing identified using DESeq2 analysis; **Supplementary Figure S10.** Network visualization of significant Pearson correlations between normalized lipid abundance and ASV counts; Gas chromatography mass spectrometry (GC-MS) methods; **Supplementary Information references**.

## Data Availability

Soil lipidome mass spectrometry post-processed lipid feature data along with amplicon sequencing 16S rRNA gene/ITS region raw and post-processed data have been deposited at the PNNL DataHUB project data repository and are available for download under the project dataset “WA-Omics_LA.1.0” accession: 10.25584/WAOmicsLA1/1866788. Primary raw mass spectrometry lipidomics and metabolomics data have been deposited at MassIVE under the accession: MSV000086931. Primary raw sequencing data has been deposited at the Sequence Read Archive (SRA) SRP400851 under the BioProject accession: PRJNA886747. The data package described in this paper is the first version. Comprehensive data package contents including amplicon 16S rRNA gene/ITS region sequencing processed data files, environmental sample metadata (MIMS.me.soil.5.0), and supporting analysis and software related to the reproducibility of the reported data have been provided at the publication data DOI page.
