# Supplementary material for: Rapid remodeling of the soil lipidome in response to a drying-rewetting event
Source: Microbiome. 2023 Feb 27;11:34. doi: 10.1186/s40168-022-01427-4 (PMC9969633; doi:10.1186/s40168-022-01427-4)
Supplement: Supplementary file 3 — Additional file 2: Supplementary Figure S1. Relative distribution of fatty acid chains; Unknown features in the soil lipidome; Statistical analysis on all lipid features (including unidentified); Supplementary Figure S2. Lipidomics PCA analysis; Supplementary Figures S3 and S4. Volcano plots for lipidomics positive and negative mode data; Classical molecular networking using GNPS; Supplementary Figures S5-S7. Molecular networks; Supplementary Figures S8 and S9. Differentially abundant ASVs in 16S rRNA and ITS region amplicon sequencing identified using DESeq2 analysis; Supplementary Figure S10. Network visualization of significant Pearson correlations between normalized lipid abundance and ASV counts; Gas chromatography mass spectrometry (GC-MS) methods; Supplementary Information references. [file 40168_2022_1427_MOESM2_ESM.pdf]

Supplementary Information for

Title: Rapid remodeling of the soil lipidome in response to a drying-rewetting event

Sneha P. Couvillion[1], Robert E. Danczak[1], Dan Naylor[1], Montana L. Smith[1], Kelly G. Stratton[1], Vanessa L. Paurus[1], Kent J. Bloodsworth[1],Yuliya Farris[1], Darren J. Schmidt[1], Rachel E. Richardson[2], Lisa M. Bramer[1], Sarah J. Fansler[1], Ernesto S. Nakayasu[1], Jason E. McDermott[1,3], Thomas O. Metz[1], Mary S. Lipton[1], Janet K. Jansson[1], Kirsten S. Hofmockel[1,4,*]

[1]Earth and Biological Sciences Directorate, Pacific Northwest National Laboratory, Richland, WA, USA

[2]National Security Directorate, Pacific Northwest National Laboratory, Richland, WA, USA

[3]Department of Molecular Microbiology and Immunology, Oregon Health & Science University, Portland, OR, USA

[4]Department of Agronomy, Iowa State University, Ames, IA, USA

*Corresponding Author: kirsten.hofmockel@pnnl.gov

This PDF file includes:

- Supplementary text
- Supplementary Figures S1 to S10
- SI References

Other supplementary materials for this manuscript include the following:

- Tables S1 to S9 in an Excel sheet

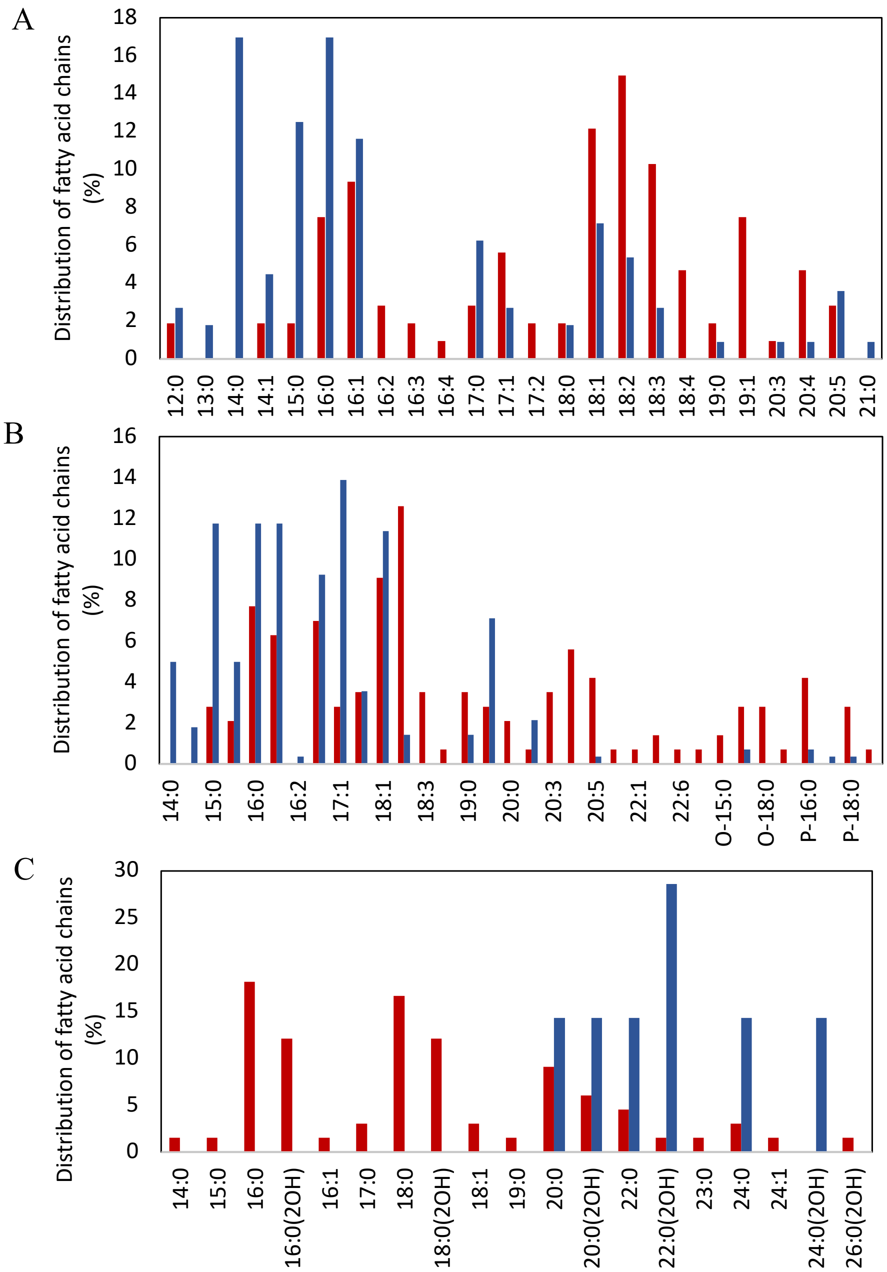

Figure S1. Relative distribution of fatty acid chains in (A) glycerolipids, (B) glycerophospholipids and (C) sphingolipids in lipids that are significantly higher in dry (0 min; red) versus wet (10-180 min; blue) soils

Unknown features in the soil lipidome

Statistical analysis of all features, known and unknown, indicated that 497 features significantly changed in abundance (See **Supplementary Figures S2-S4**). This finding suggests that the unknown features could provide valuable biological insights if they could be identified. We deployed molecular networking (1) to uncover unknown molecules that are structurally related to known lipids (**Supplementary Figures S5-S7**). Molecular networking enables the detection and visualization of similar spectra from structurally related molecules with similar fragmentation patterns. Searching the MS/MS data against reference GNPS (Global Natural Products Social Molecular Networking) spectral libraries resulted in putative matches to some interesting non-lipid metabolites that might have partitioned into the organic layer during sample extraction. These included cyclic peptides (thermoactinoamides) and chlorophyll related molecules.

Statistical analysis on all lipid features (including unidentified)

For feature detection and quantification, LC-MS/MS data in positive and negative mode was processed separately, using a method similar to the feature-based molecular network described previously (2). Raw files were converted to mzXML format and imported into MZmine 2 (3). The mass detection was performed on mass level 1 and mass level 2, by keeping the noise level at 1.5E4 and 2.0E3 respectively for positive mode data and 1.5E4 and 2.0E1 respectively for the negative mode data. Chromatograms were built using an ADAP module (4) with an *m/z* tolerance of 5 ppm and a minimum height of 15E3 for positive mode data and 1.0E5 for negative mode data respectively. For the chromatogram deconvolution, the baseline cut-off algorithm was used with the following settings for positive mode data: min. peak height=1.0E5, peak duration range (min)=0.01 to 3.0, baseline level= 1.0E3, m/z range for MS2 scan pairing (Da)= 0.02, RT range for MS2 scan pairing (min)= 0.15. For negative mode data, the following settings were used: min. peak height=1.0E4, peak duration range (min)=0.01 to 3.0, baseline level= 1.0E2, m/z range for MS2 scan pairing (Da)= 0.02, RT range for MS2 scan pairing (min)= 0.15.

The isotopic peak grouper was used to group isotope and co-eluting ions using m/z tolerance of 5 ppm and retention time tolerance= 0.15 min, maximum charge=3.Peak alignment was performed using the Join aligner algorithm: *m/z* tolerance at 5 ppm (weight=75), absolute RT tolerance at 0.15 min (weight=25). Features not present in at least 3 datasets, those that did not have at least 2 peaks in an isotope pattern and those without associated MS/MS spectrum were filtered out from the peak list. Gap filling was done with an intensity tolerance of 10%, *m/z* tolerance of 20 ppm and retention time tolerance of 0.2 min. Additional peak filtering was done on the gap-filled list to only include peaks with a duration between 0.05 to 0.6 min and height range of 1.0E5 to 1.0E10. Feature quantification data including *m/z*, retention times, peak heights were exported to a .csv file for downstream statistical analysis.

The negative lipidomics data contained 690 lipids and 252 missing values. The positive lipidomics data contained 963 lipids and 217 missing values. There are various reasons for a lipid abundance to be missing in an untargeted lipidomics dataset. The lipid species can be truly absent in the sample or may be below the limit of detection. The missing values in the data were left as is and not imputed. A minimum of 2 observations per group were required for the downstream statistical

comparisons. A robust Mahalanobis distance based on lipid abundance vectors (rMd-PAV) was calculated to identify potential sample outliers in the data (5) but no outliers were found.

Plotting samples by the first two principal components showed some clustering of the time 0 min samples (dry) apart from the later time point samples (wet) for both the positive (**Fig. S2(A)**) and negative (**Fig. S2(B)**) lipidomics data. Normalization was done using global median centering. Statistical comparisons (ANOVA with a Dunnett test correction) of each later time point (after the soil was wetted) back to the time 0 group (which corresponded to dry soil) were performed. Volcano plots in **Fig. S3** and **Fig. S4** show the -log10 adjusted p-values against log2 fold change for all positive and negative mode lipids. Those above the dashed line were found to be significant (p-value<0.05).

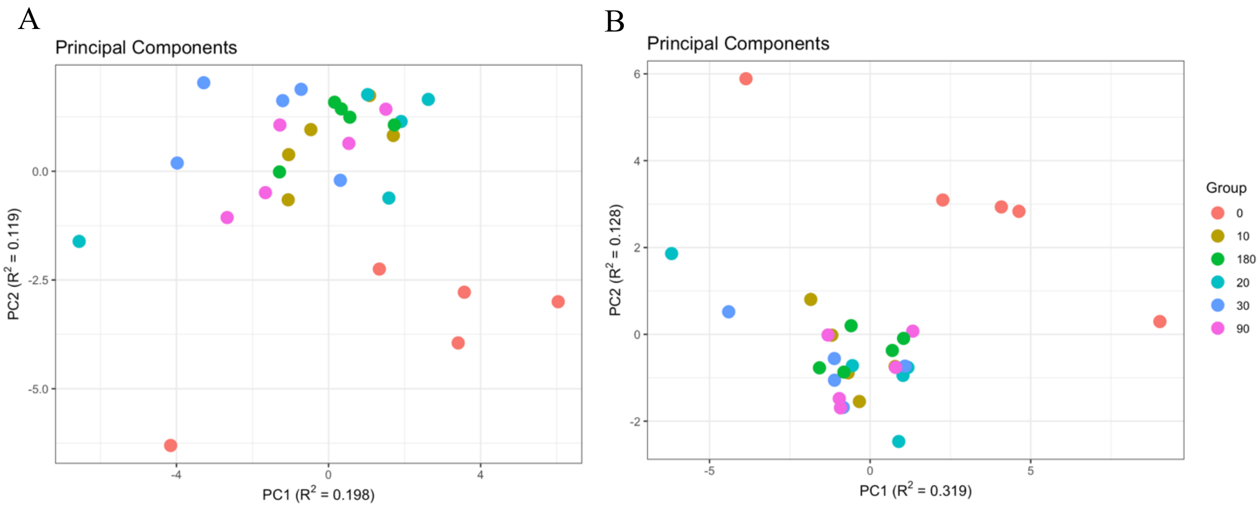

Figure S2. Samples by first two principal components, colored by time, for the **(A)** positive and **(B)** negative lipidomics data

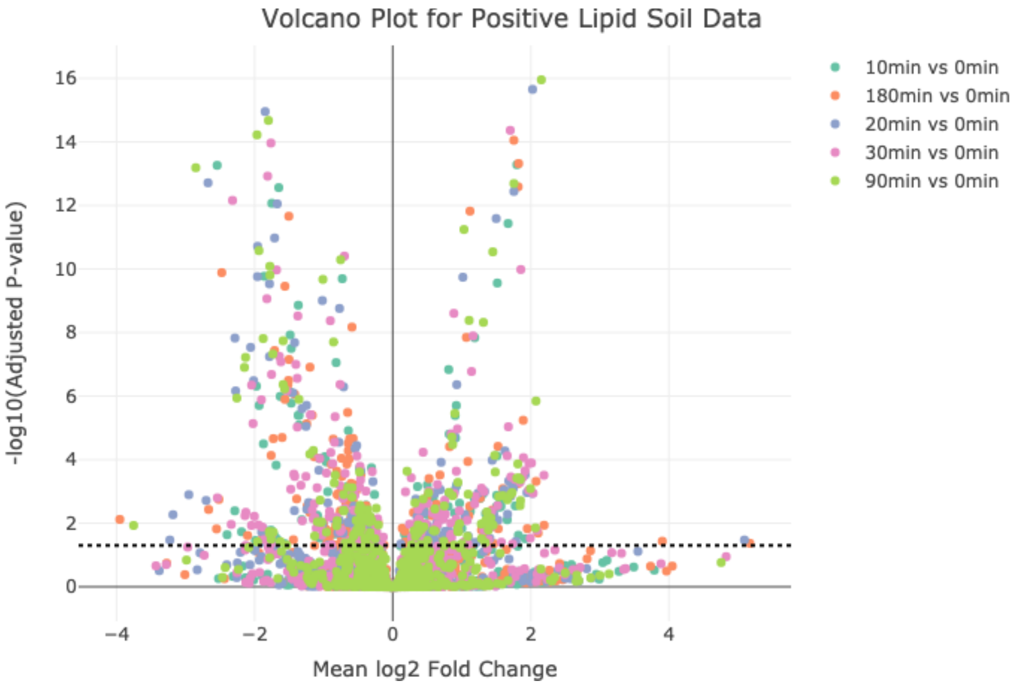

Figure S3. -log10 adjusted p-values against log2 fold change for all positive mode lipids. Those above the dashed line were found to be significant.

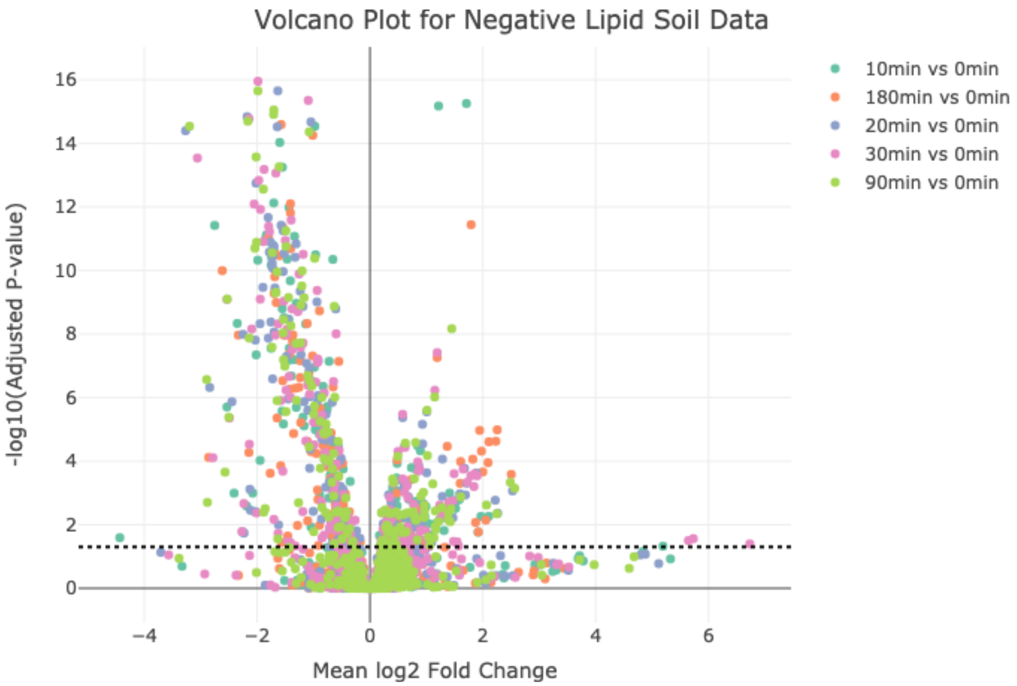

Figure S4. -log10 adjusted p-values against log2 fold change for all negative mode lipids. Those above the dashed line were found to be significant.

Classical molecular networking using GNPS

Molecular networks (1) were created separately for data from positive (https://gnps.ucsd.edu/ProteoSAFe/status.jsp?task=dae3199f852c45d994f4c777d7399701) and negative(https://gnps.ucsd.edu/ProteoSAFe/status.jsp?task=f9e562588c4244e7a2b1bebf80a8ec6f) ionization modes using the online workflow (https://ccms-ucsd.github.io/GNPSDocumentation/) on the GNPS website (http://gnps.ucsd.edu). The data was filtered by removing all MS/MS fragment ions within +/- 17 Da of the precursor m/z. MS/MS spectra were window filtered by choosing only the top 6 fragment ions in the +/- 50Da window throughout the spectrum. The precursor ion mass tolerance was set to 2.0 Da and a MS/MS fragment ion tolerance of 0.5 Da. A network was then created where edges were filtered to have a cosine score above 0.7 and more than 4 matched peaks. Further, edges between two nodes were kept in the network if and only if each of the nodes appeared in each other's respective top 10 most similar nodes. Finally, the maximum size of a molecular family was set to 100, and the lowest scoring edges were removed from molecular families until the molecular family size was below this threshold. The spectra in the network were then searched against GNPS' spectral libraries. The library spectra were filtered in the same manner as the input data. All matches kept between network spectra and library spectra were required to have a score above 0.7 and at least 4 matched peaks. The networks were visualized using Cytoscape (6) 3.8.0. Only the first neighbor nodes of the identified nodes have been included in the figures.

Fig. S5 shows a molecular family containing nodes putatively identified as thermoactinoamides A, B, E and H. Thermoactinoamides are lipophilic cyclic hexapeptides that were recently isolated from a thermophilic bacterium *Thermoactinomyces vulgaris* which was grown at 60°C (7). Cyclic peptides are bioactive natural products that often possess anti-microbial and anti-tumor properties (8). This indicates that the soil environment contains microbes that produce thermoactinoamides and suggests the possible role of these cyclopeptides in microbial adaptation to hot and arid environments. **Fig. S6** shows a molecular family with nodes that have putative identifications of chlorophyll and chlorophyll derivates pheophytin, pyropheophytin and bacteriopheophytin. Pheophytins are chlorophyll molecules lacking the Mg ion. Like chlorophyll, they play important roles in photosynthetic orgamisms. The presence of the molecules indicates the presence of photosynthetic microbes in the soil. **Fig. S7** shows a molecular family containing nodes putatively identified as ceramides which are sphingolipids.The presence of putatively identified MS/MS spectra (blue nodes) in a spectral family provide a useful starting point for identification of unannotated MS/MS spectra (red nodes) in each of these families.

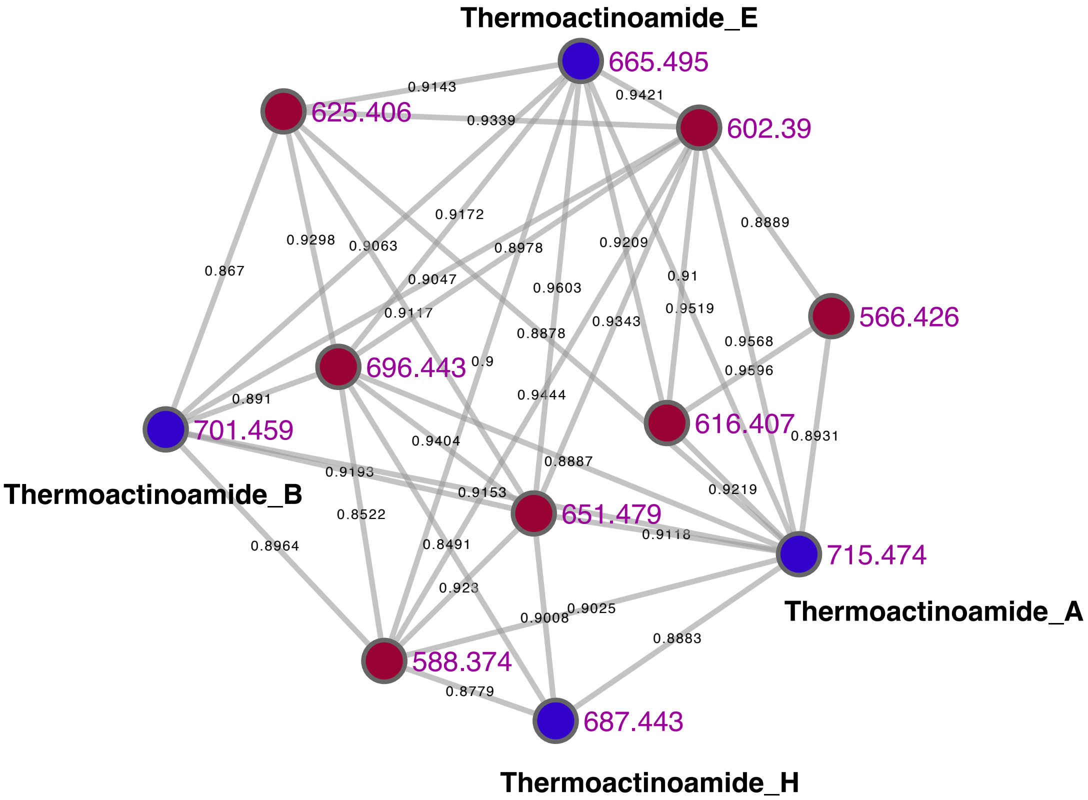

Figure S5. Molecular network showing a molecular family of thermoactinoamides and related molecules. Four nodes were putatively identified using GNPS library matching. Nodes with a GNPS library match are labelled blue and unannotated nodes are labelled red. The parent mass is indicated in purple and the cosine similarity scores of the edges are in black.

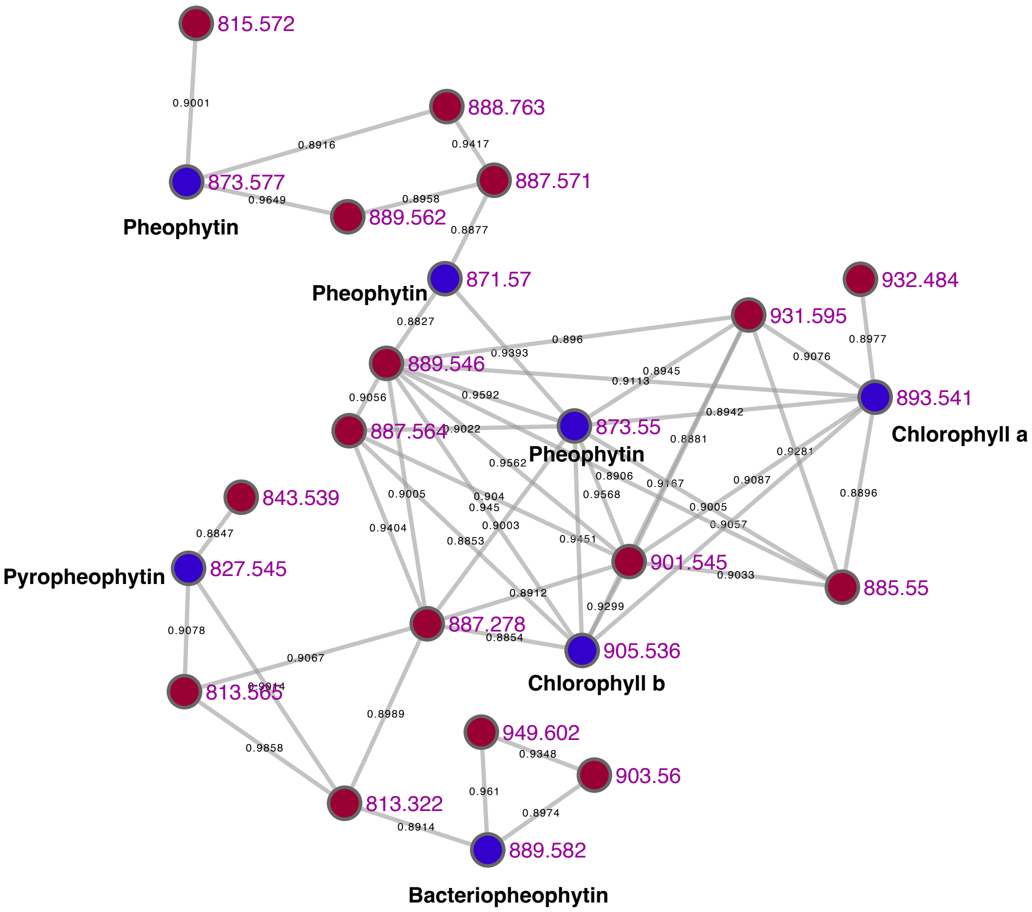

Figure S6. Molecular network showing a molecular family of chlorophyll and related molecules. Nine nodes were putatively identified using GNPS library matching. Nodes with a GNPS library match are labelled blue and unannotated nodes are labelled red. The parent mass is indicated in purple and the cosine similarity scores of the edges are in black.

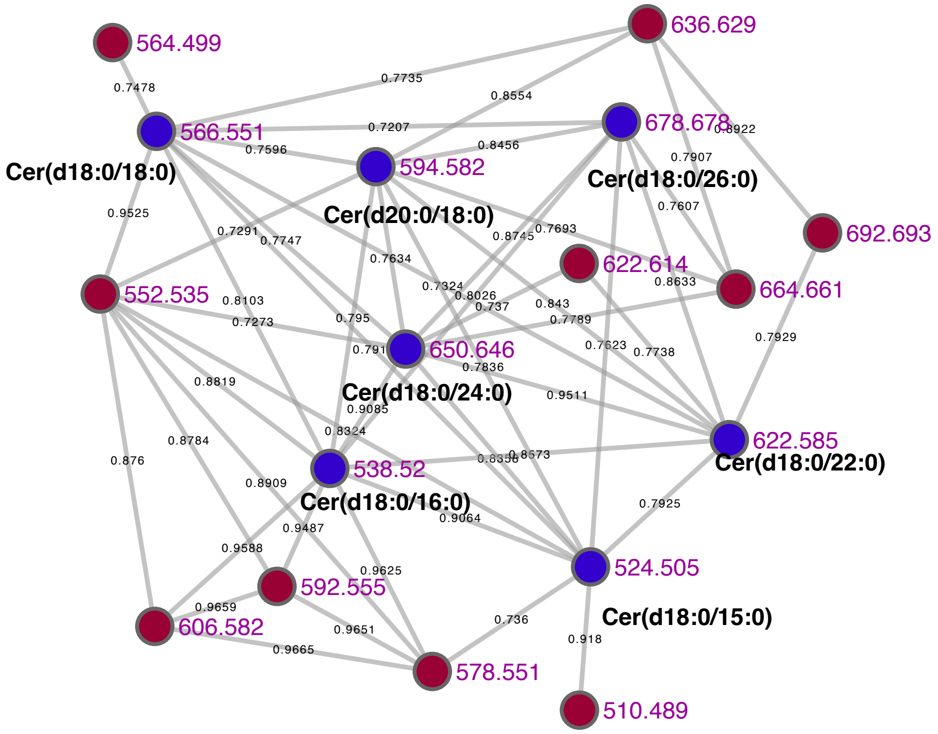

Figure S7. Molecular network showing a molecular family of ceramides and related molecules. Seven nodes were putatively identified using GNPS library matching. Nodes with a GNPS library match are labelled blue and unannotated nodes are labelled red. The parent mass is indicated in purple and the cosine similarity scores of the edges are in black.

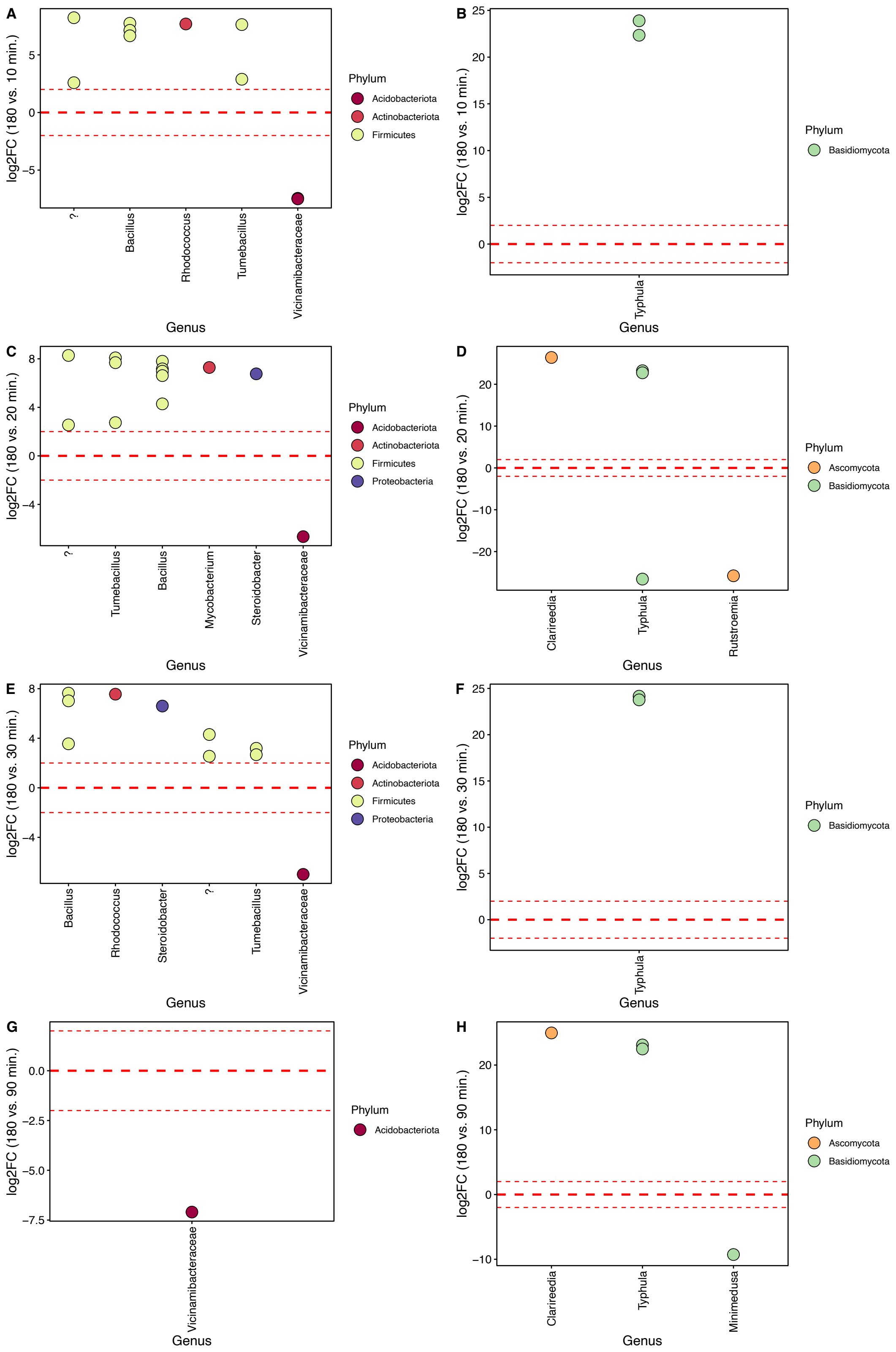

Figure S8. Differentially abundant ASVs between final wet (180 min) and earlier wet (10, 20, 30 or 90 min) timepoints identified through DESeq2 analysis. ASVs are color-coded according to their phylum.

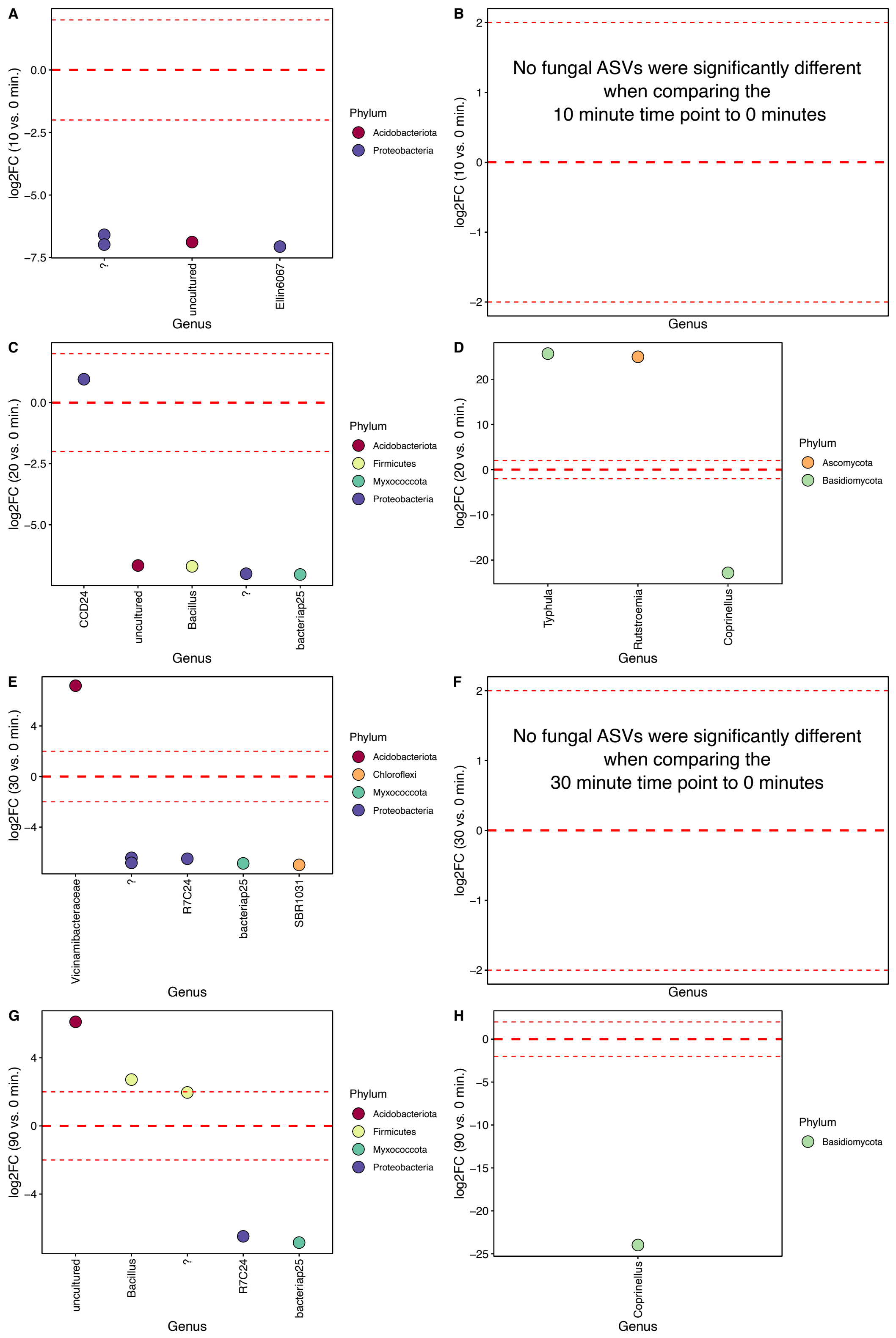

Figure S9. Differentially abundant ASVs between dry (0 min) and wet (10, 20, 30 or 90 min timepoints) soil identified through DESeq2 analysis. ASVs are color-coded according to their phylum.

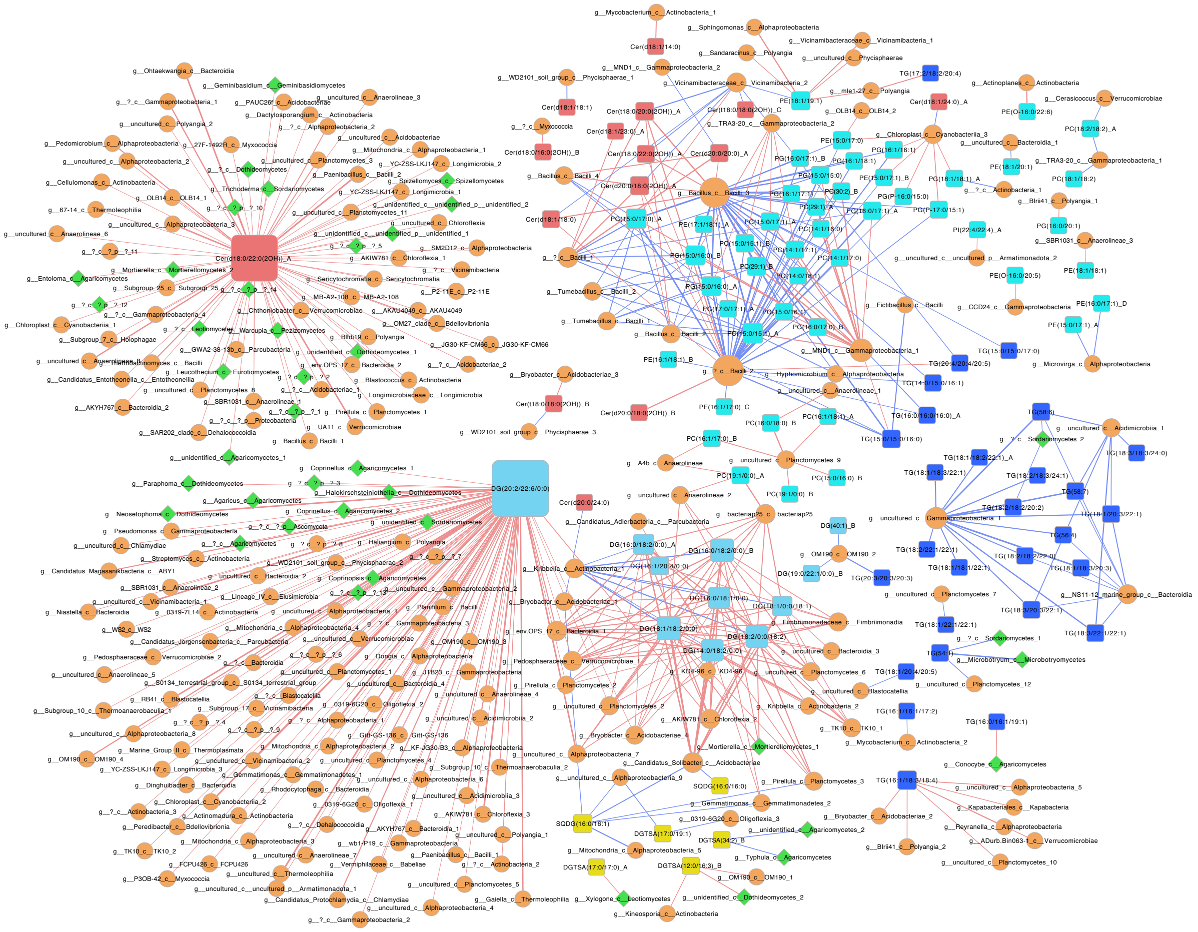

Figure S10. A network visualization of significant Pearson correlations between normalized lipid abundance (squares) and ASV counts from rarefied 16S rRNA gene (orange circles) and ITS region (green diamonds) amplicon data. Edge thickness is proportional to the correlation coefficient with blue edges showing positive correlations and red edges showing negative correlations. Node size is proportional to the number of connected edges. Lipid node colors: glycerophospholipids - cyan, TGs - dark blue, DGs - light blue and phosphorus-free betaine and SQDG lipids - yellow. Bacterial and fungal nodes are labelled to indicate genus if available and class.

Gas chromatography mass spectrometry (GC-MS):

Dried extracts were chemically derivatized using a modified version of the protocol used to create FiehnLib (9). Briefly, dried metabolite extracts were dried again to remove any residual water from being stored at −80 °C. To protect carbonyl groups and reduce the number of tautomeric isomers, 20 µl of methoxyamine in pyridine (30 mg ml^{-1}) was added to each sample, followed by vortexing for 30 s and incubation at 37 °C with vigorous shaking (1,000 r.p.m.) for 90 min. The sample vials were then inverted once to capture any condensation of solvent at the cap surface, followed by a brief centrifugation at 1,000 × g for 1 min. To derivatize hydroxyl and amine groups to trimethylsilylated (TMS) forms, 80 µl of N-methyl-N-(trimethylsilyl)trifluoroacetamide (MSTFA) with 1% trimethylchlorosilane (TMCS) were then added to each vial, followed by vortexing for 10 s and incubation at 37 °C with shaking (1,000 r.p.m.) for 30 min. Again, the sample vials were inverted once, followed by centrifugation at 1,000 × g for 5 min. The samples were allowed to cool to room temperature and analyzed the same day.

An Agilent GC 7890A coupled with a single quadrupole MSD 5975C (Agilent Technologies) was used and the samples were analyzed in random order for each experiment. An HP-5MS column (30 m × 0.25 mm × 0.25 µm; Agilent Technologies) was used for untargeted metabolomics analyses. The sample injection mode was splitless and 1 µl of each sample was injected. The injection port temperature was held at 250 °C throughout the analysis. The GC oven was held at 60 °C for 1 min after injection and the temperature was then increased to 325 °C by 10 °C min^{-1}, followed by a 5 min hold at 325 °C (10). The helium gas flow rates for each experiment were determined by the Agilent Retention Time Locking function based on analysis of deuterated myristic acid and were in the range of 0.45–0.5 ml min^{-1}. Data were collected over the mass range 50–550 *m/z*. A mixture of fatty acid methyl esters (FAMEs) (C8–C28) was analyzed once per day together with the samples for retention index alignment purposes during subsequent data analysis.

GC-MS raw data file processing was done using Metabolite Detector software and metabolites were identified by matching experimental spectra and retention indices to an augmented version of FiehnLib (9). All identifications were manually validated to reduce deconvolution errors and to eliminate false identifications. The NIST 14 GC–MS library was also used to cross-validate the spectral matching scores obtained using the Agilent library and to provide identifications of unmatched metabolites.

SI References

1. M. Wang *et al.*, Sharing and community curation of mass spectrometry data with Global Natural Products Social Molecular Networking. *Nature Biotechnology* **34**, 828-837 (2016).
2. L.-F. Nothias *et al.*, Feature-based molecular networking in the GNPS analysis environment. *Nature Methods* **17**, 905-908 (2020).
3. T. Pluskal, S. Castillo, A. Villar-Briones, M. Oresic, MZmine 2: modular framework for processing, visualizing, and analyzing mass spectrometry-based molecular profile data. *BMC Bioinformatics* **11**, 395 (2010).
4. O. D. Myers, S. J. Sumner, S. Li, S. Barnes, X. Du, One Step Forward for Reducing False Positive and False Negative Compound Identifications from Mass Spectrometry Metabolomics Data: New Algorithms for Constructing Extracted Ion Chromatograms and Detecting Chromatographic Peaks. *Analytical Chemistry* **89**, 8696-8703 (2017).
5. M. M. Matzke *et al.*, Improved quality control processing of peptide-centric LC-MS proteomics data. *Bioinformatics* **27**, 2866-2872 (2011).
6. P. Shannon *et al.*, Cytoscape: A Software Environment for Integrated Models of Biomolecular Interaction Networks. *Genome Research* **13**, 2498-2504 (2003).
7. R. Teta *et al.*, Thermoactinoamide A, an Antibiotic Lipophilic Cyclopeptide from the Icelandic Thermophilic Bacterium Thermoactinomyces vulgaris. *Journal of Natural Products* **80**, 2530-2535 (2017).
8. B. Behsaz *et al.*, De Novo Peptide Sequencing Reveals Many Cyclopeptides in the Human Gut and Other Environments. *Cell Systems* **10**, 99-108.e105 (2020).
9. T. Kind *et al.*, FiehnLib: mass spectral and retention index libraries for metabolomics based on quadrupole and time-of-flight gas chromatography/mass spectrometry. *Anal Chem* **81**, 10038-10048 (2009).
10. S. Khare *et al.*, Effects of ingested nanocellulose on intestinal microbiota and homeostasis in Wistar Han rats. *NanoImpact* **18**, 100216 (2020).
